# The Small GTPase MoSec4 Is Involved in Vegetative Development and Pathogenicity by Regulating the Extracellular Protein Secretion in *Magnaporthe oryzae*

**DOI:** 10.3389/fpls.2016.01458

**Published:** 2016-09-27

**Authors:** Huakun Zheng, Simiao Chen, Xiaofeng Chen, Shuyan Liu, Xie Dang, Chengdong Yang, Martha C. Giraldo, Ely Oliveira-Garcia, Jie Zhou, Zonghua Wang, Barbara Valent

**Affiliations:** ^1^State Key Laboratory of Ecological Pest Control for Fujian and Taiwan Crops, College of Life Science, Fujian Agriculture and Forestry UniversityFuzhou, China; ^2^Department of Plant Pathology, Kansas State UniversityManhattan, KS, USA; ^3^Basic Forestry and Proteomics Research Center, Haixia Institute of Science and Technology (HIST), Fujian Agriculture and Forestry UniversityFuzhou, China

**Keywords:** Rab GTPase, exocyst complex, MoSec4, effector secretion, *Pyricularia oryzae*

## Abstract

The Rab GTPase proteins play important roles in the membrane trafficking, and consequently protein secretion and development of eukaryotic organisms. However, little is known about the function of Rab GTPases in *Magnaporthe oryzae*. To further explore the function of Rab GTPases, we deleted the ortholog of the yeast Sec4p protein in *M. oryzae*, namely *MoSEC4*. The Δ*Mosec4* mutant is defective in polarized growth and conidiation, and it displays decreased appressorium turgor pressure and attenuated pathogenicity. Notably, the biotrophic invasive hyphae produced in rice cells are more bulbous and compressed in the Δ*Mosec4* mutant. Further studies showed that deletion of the *MoSEC4* gene resulted in decreased secretion of extracellular enzymes and mislocalization of the cytoplasmic effector PWL2-mCherry-NLS. In accordance with a role in secretion, the GFP-MoSec4 fusion protein mainly accumulates at tips of growing vegetative hyphae. Our results suggest that the MoSec4 protein plays important roles in the secretion of extracellular proteins and consequently hyphal development and pathogenicity in the rice blast fungus.

## Introduction

*Magnaporthe oryzae* (synonym to *Pyricularia oryzae*) is the causal agent of rice blast disease that destroys almost a quarter of the potential global rice harvest every year (Talbot, 2003), and it is also a model system for understanding microbe-plant interactions. *M. oryzae* is a hemibiotrophic fungus that penetrates the plant cuticle using a pressurized appressorium, and then grows asymptomatically for about 4 days before sporulating lesions appear. During this biotrophic growth phase, specialized invasive hyphae (IH) successively colonize one living rice cell after the next, and invaded host cells die around the time IH move into neighboring cells. The blast fungus secretes hundreds of effector proteins throughout the disease cycle, including biotrophy-associated secreted (BAS) proteins that are specifically expressed during biotrophic invasion (Mosquera et al., 2009). Effectors are presumed to suppress rice cell immunity (Mentlak et al., 2012; Park et al., 2012) and otherwise facilitate infection (Saitoh et al., 2012; Chen et al., 2013; Dong et al., 2015). Effectors are divided into apoplastic effectors, which remain outside host cells, and cytoplasmic effectors, which are translocated across the host plasma membrane inside living cells. For blast disease, secreted apoplastic effectors are retained inside the extrainvasive hyphal membrane (EIHM) compartment surrounding IH growing inside rice cells, resulting in outlining of the IH (Mosquera et al., 2009; Khang et al., 2010). Cytoplasmic effectors preferentially accumulate in a specialized Biotrophic Interfacial Complex (BIC), which is proposed to be the location where translocation of cytoplasmic effectors occurs (Khang et al., 2010). Apoplastic and cytoplasmic blast effectors apparently differ in secretion pathways (Giraldo et al., 2013). Secretion of apoplastic effectors is disrupted by treatment with Brefeldin A (BFA), an inhibitor of ER-to-Golgi secretion in fungi. In contrast, secretion of cytoplasmic effectors into BICs continues in the presence of BFA, suggesting they are secreted through a golgi-independent secretion pathway. Nonconventional secretion of cytoplasmic effectors does involve the exocyst complex, since *M. oryzae* mutants lacking exocyst components MoSec5 and MoExo70 show partial retention of fluorescent cytoplasmic effector protein inside BIC-associated hyphal cells, indicating inefficient secretion (Giraldo et al., 2013).

Membrane vesicular trafficking, including exocytosis and endocytosis, is essential for development and response to the environment in filamentous fungi, as well as in other eukaryotic cells. From the budding yeast *Saccharomyces cerevisiae* to humans, Rab GTPase family proteins play conserved roles as regulators (membrane-associated molecular switches) of intracellular vesicle trafficking, including extracellular protein secretion pathways. Among them, budding yeast Rab GTPase Sec4p (Rab8 in humans) acts as a master regulator at a final stage of protein secretion (Novick, 2014). Sec4p controls vesicle trafficking from golgi to the plasma membrane by regulating the assembly and docking of the 8-subunit exocyst complex at the target point for secretion (Hsu et al., 1996; TerBush et al., 1996). The active (GTP-bound) form of Sec4 protein on the secretory vesicle recruits a partially assembled exocyst complex by binding to Sec15p, which is complexed with Sec5p, Sec6p, Sec8p, Sec10p, and Exo84p. In turn, this vesicular subcomplex docks with Sec3p and Exo70p at the target region of the plasma membrane (PM). Then the SNARE (Soluble N-ethylmaleimide-sensitive factor attachment protein receptor) proteins mediate fusion of the secretory vesicle with the PM (He and Guo, 2009). Increasing evidence supports the conserved role of Sec4 Rab GTPase orthologs as a key regulator in the secretion of extracellular proteins in a wide range of organisms including filamentous fungi (Punt et al., 2001; Powell and Temesvari, 2004; Siriputthaiwan et al., 2005; Zhang et al., 2014).

Among the 11 predicted Rab GTPase proteins in *M. oryzae*, MoYpt7 is required for membrane fusion in autophagy and pathogenicity (Liu et al., 2015). Additionally, MoRab5A and MoRab5B may perform non-redundant functions in endosomal sorting (Qi et al., 2014). However, the role of the Sec4 ortholog in *M. oryzae* remains unknown. MoSec4 function is of special interest based on its key role in regulating exocyst function in *S. cerevisiae*, and the special role of the exocyst in the secretion of cytoplasmic effectors in *M. oryzae* (Giraldo et al., 2013). Here, we report that the MoSec4 protein is essential for normal growth and morphological development both on agar media and in planta. Both secretion of extracellular enzymes and pathogenicity are diminished in the Δ*Mosec4* mutants. Although the mutants do appear to be impaired in localization of secreted cytoplasmic effectors, fluorescent effector protein is not observed backed up inside BIC-associated cells as occurs during impaired effector secretion in Δ*Moexo70* and Δ*Mosec5* exocyst component mutants. Thus, we find no evidence to support a specific role for MoSec4 in the nonconventional, exocyst-assisted pathway for secretion of cytoplasmic effectors into BICs. Instead it appears that a general secretion defect impacts fungal growth, morphological development, and pathogenicity.

## Materials and methods

### Fungal strains and manipulations

*M. oryzae* wild-type strain Guy11 and the parental strain “Ku80,” which is wild type Guy11 with a deletion of *MoKU80* gene to enhance homologous recombination, were used as control strains throughout this study (Villalba et al., 2008). All strains were stored dried and frozen in cellulose filter paper as described (Valent et al., 1991). For conidiation, strains were cultured on rice bran medium containing 2% rice bran and 1.5% agar (pH 6.0). For testing the utilization of different carbon sources, strains were grown on minimal medium (MM) (Foster et al., 2003) with 10 g/L glucose or an equivalent amount of sucrose, starch or chitin as the sole carbon source. For testing response to chemical stresses, strains were grown on complete medium (CM) (Foster et al., 2003) containing 300 mg/L Congo red, 7.5 mmol/L H_2_O_2_, 0.01% SDS, or 200 μg/mL Calcofluor white (CFW). For all the plate growth assays, similarly-sized plugs of each strain were transferred from starch-yeast medium (SYM) to the appropriate media plates, and plates were imaged after cultivation for 10 days. All assays in this study were performed in three independent biological experiments with at least three replicates. The significance analysis was performed using the Duncan's multiple range test in SPSS software (Bryman and Cramer, 2012). This method was applied in all the other statistic analyses except for the width/length ratio of IH in **Figure 5** and the optical density/g mycelia in **Figure 7C**.

### Targeted gene deletion and complementation of *MoSEC4*

To generate Δ*Mosec4* mutants, a 616-bp upstream fragment (A) and a 741-bp downstream fragment (B) of the targeted ORF were amplified from the *M. oryzae* genome using the primer sets *MoSEC4*-2F and *MoSEC4*-2R, and *MoSEC4*-3F and *MoSEC4*-3R, respectively (Figure S1 and Table S3). The purified products were inserted into the *Hin*d III—*Eco*R I and *Bam*H I—*Spe* I sites of plasmid pCX62 that contained the hygromycin B phosphotransferase cassette (H). The “A-H-B” fusion was amplified from pG06135KO (Table S2) using primers *MoSEC4*-2F and *MoSEC4*-3R (Table S3) and transformed into the protoplasts of strain Ku80 through PEG-mediated transformation as described previously (Sweigard et al., 1992, 1998). To identify the targeted gene deletion mutants, all hygromycin-resistant transformants were first screened by PCR using primers *MoSEC4*-4F and *MoSEC4*-4R, and *MoSEC4*-5F and *MoSEC4*-5R (Table S3). The positive transformants were confirmed through Southern hybridization and RT-PCR.

For complementation of Δ*Mosec4* mutant, a 2.2-kb genomic DNA fragment containing the native promoter, CDS region and 3′-UTR of *MoSEC4*, was amplified using primers *MoSEC4*-6F and *MoSEC4*-6R (Table S3), and cloned into pKNTG plasmid. The resulting construct was reintroduced into Δ*Mosec4* mutant through PEG-mediated transformation. The neomycin-resistant transformants were evaluated through RT-PCR and Southern hybridization.

To produce the *P*_*MoSEC*4_*:GFP:MoSEC4* transformants for complementation and fusion protein localization, the pBV1171 (Table S2) was introduced into the Δ*Mosec4* mutant and the transformants were evaluated through PCR-based genotyping using *GFPXbaIF-MoSEC4tR* and *MoSEC4ptF-MoSEC4tR* primer sets (Table S3).

### Southern hybridization assay

5–10 μg genomic DNA of each strain was completely digested with *Eco*RI. Digested products were electrophoresed in a 1% (m/v) agarose gel and then transferred onto a Hybond-N^+^ membrane (Amersham Pharmacia Biotech Inc.). The specific hybridization probe was amplified using primers *MoSEC4*-4F and *MoSEC*-4R (Table S3). Probe labeling, hybridization and detection were performed using DIG High Prime DNA Labeling and Detection Starter Kit I (Roche Applied Science), according to the instruction manual.

### Physiological assays

After the removal of aerial mycelia, culture blocks (4 mm^2^) were cut from 5-day-old rice bran agar cultures, laid on their sides on slides and placed into moisturizing plastic plates. After the induction of conidiogenesis for 24 h in a chamber with constant-light conditions, conidial development was observed using an Olympus BX53 microscope (Liu et al., 2010).

To analyze the conidial morphology, conidia of each strain collected from 10-day-old rice bran agar cultures were quantified using a hemocytometer, and were observed for morphology under light microscopy. The length and width of spores and IH cells were measured using the line tool of ZEN 2010 software (Zeiss).

To examine conidial germination and appressorial formation, 10 μL of conidial suspension (5 × 10^4^ conidia/mL) were dropped on the hydrophobic side of Gelbond film (Lonza), and then incubated in a moist chamber at 28°C. The percentages of conidial germination and appressorial formation were determined by microscopic examination at 4, 8, 12 hpi.

The cytorrhysis assay was performed for measuring appressorium turgor as described (de Jong et al., 1997). In brief, conidia were incubated on the surface of artificial hydrophobic Gelbond film, and treated with different concentrations of glycerol (1M, 2M, 3M) after appressorium formation (at 24 h post germination). Collapsed, sometimes also ruptured, appressoria were counted using DIC microscopy 5 min after glycerol treatment.

### Plant infection assay

Conidial suspensions (1 × 10^5^ conidia/mL in 0.25% gelatin solution) of each strain were used for inoculation assays on 15-day-old rice seedlings of susceptible rice varieties CO39 and YT16. Rice seedlings were cultivated under the conditions described previously (Giraldo et al., 2013). Plant inoculation and subsequent incubation were performed as previously described, with the humidity maintained below 90% to block conidation (Valent et al., 1991). Lesion formation was examined at 7 days post inoculation. Lesion types were defined according to Valent et al. (1991). Specifically, Type 0 indicated no visible evidence of infection; type 1, uniform dark brown pinpoint lesions up to 0.5 mm long without visible centers; type 2, small lesions with distinct tan/green centers surrounded by a darker brown margin (up to 1 mm); type 3, small eyespot lesions ~1.5 mm in length with tan centers surrounded by dark brown margins; type 4, intermediate size eyespot lesions, up to 2 mm in length; type 5, large eyespot lesions that attain the maximum size characteristic for a particular cultivar/strain interaction (~3 mm in length for Guy11 on C039). Lesion numbers were counted using 5 cm-long diseased rice blade sections taken from the youngest leaves at the time of inoculation. Assays were repeated at FAFU and KSU with similar results.

Leaf sheath inoculation was performed using 4–5 week old rice plants as described (Kankanala et al., 2007). For the penetration rate assay, the spore solutions (5 × 10^4^ conidia/mL) were inoculated into the leaf sheath of susceptible rice line YT16 as described (Giraldo et al., 2013) and examined at 28 h after inoculation.

### Survey of the extracellular enzyme activities

A 3 × 3 mm hyphal tip plug was inoculated into fresh CM liquid media and incubated at 28°C, 150 rpm for 3 days. Mycelia were completely removed by filtration, and the culture filtrates were used for the measurement of extracellular enzyme activities. Laccase activity was measured using the ABTS method as described previously (Song et al., 2010). The activities of amylase, invertase, and cellulase were determined using the DNS method as described (Miller, 1959). The dry weights of the harvested mycelia were measured for normalizing the enzyme activities.

### Live-cell imaging assay

The live-cell imaging assay was performed as described previously (Giraldo et al., 2013). For vegetative hyphal imaging, a small plug of mycelium was cut from an agar culture, placed on the edge of a sterile water agar-coated microscope slide and incubated in a humid chamber for 16–18 h. For imaging invasive hyphae, leaf sheath inoculation was performed using the susceptible rice line YT16. Differential interference contrast microscopy was performed with a Zeiss Axioplan 2 IE MOT microscope, using × 40/0.75 and × 63/1.2 NA (numerical aperture) C-Apochromat water immersion objective lens. Images were acquired using a Zeiss AxioCam HRc camera and analyzed with Zeiss Axiovision digital image-processing software (version 4.8). Confocal imaging was performed with a Zeiss LSM780 confocal microscope system using two water immersion objectives, C-Apochromat 40x/1.2 W Corr and C-Apochromat 40x/1.2 W Corr. Images were acquired and processed using ZEN 2010 software.

## Results

### Identification of the Sec4 ortholog from *M. oryzae*

**Figure 1:**
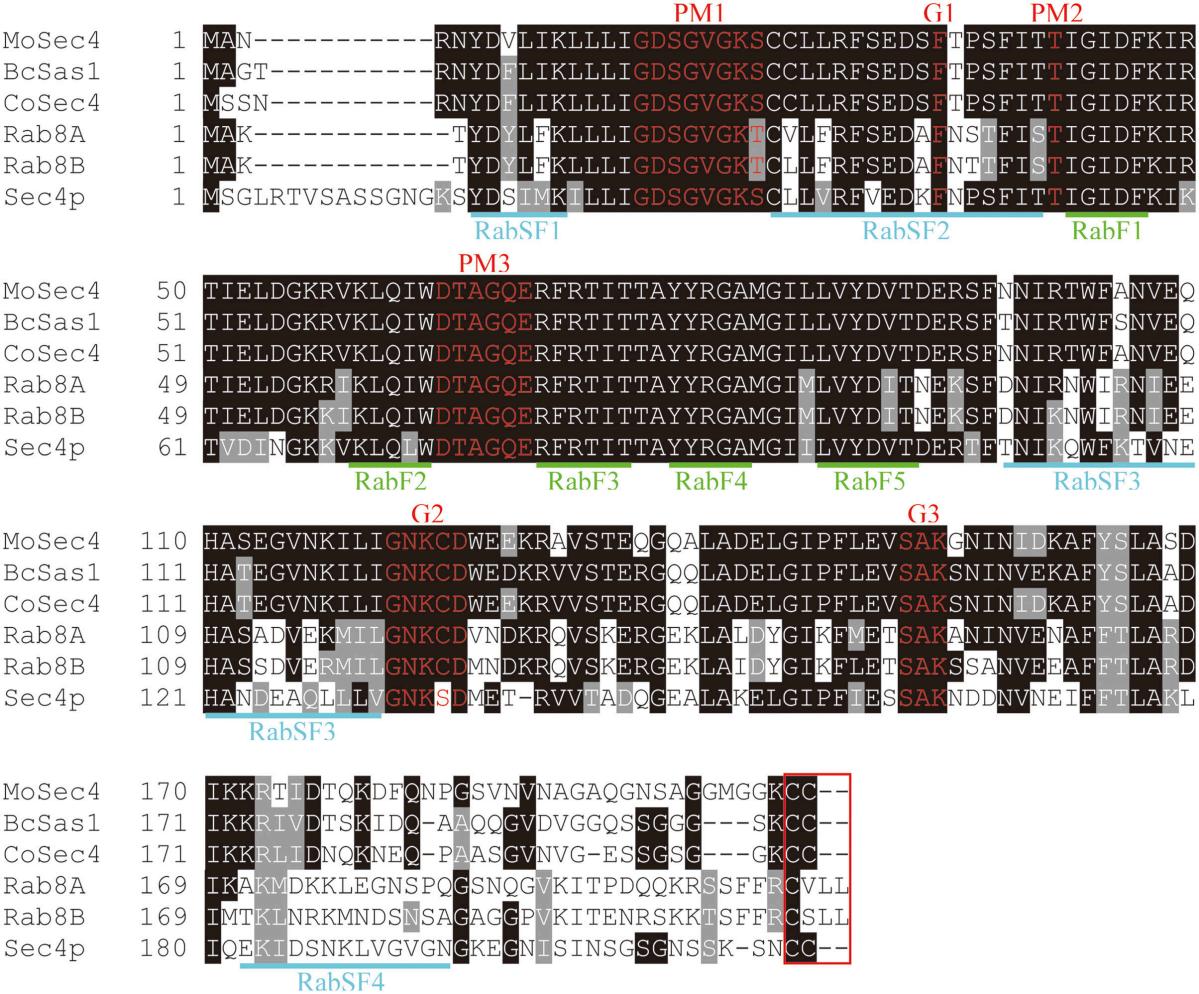
**MoSec4 is a highly conserved Rab protein**. Sequence alignment of MoSec4 with BcSas1 in *B. cinerea*, CoSec4 in *C. orbiculare*, Sec4p in yeast, and Rab8A and Rab8B in humans. Five Rab-specific motifs, RabF1-F5, were marked in green. Four subfamily-specific motifs RabSF1-SF4 were marked in blue. Highly conserved guanine (G) and phosphate/Mg^2+^ (PM) binding motifs were highlighted in red. The C-terminal motif related to protein localization was boxed in red.

To identify the Sec4 ortholog in *M. oryzae*, the amino acid sequence of Sec4p from the *S. cerevisiae* genome database (http://www.yeastgenome.org/) was used for a BLAST search in the *M. oryzae* genome database (http://www.broadinstitute.org/ annotation/genome/magnaporthe_grisea/MultiHome.html). This search identified MGG_ 06135.7, termed MoSec4, as the ortholog of *S. cerevisiae* Sec4p. The MoSec4 protein shares high amino acid identity with its orthologs, such as CoSec4 in *Colletotrichum orbiculare* (BAO27795.1, 89% identity), BcSas1 in *Botrytis cinerea* (BC1G_14039.1, 87% identity), Sec4p in *S. cerevisiae* (NP_116650.1, 64% identity), and both Rab8A (NP_005361.2, 65% identity) and Rab8B (NP_057614.1, 63% identity) in *Homo sapiens*. Multiple sequence alignment indicated that all the above six proteins possess the signature motifs of Rab GTPases, including highly conserved Rab-specific residues RabF1-F5, Rab subfamily-specific sequences RabSF1-SF4, highly conserved motifs involved in guanine and phosphate/Mg^2+^ binding (G1-G3 and PM1-PM3), and a cysteine motif at carboxyl terminus required for subcellular localization (Figure 1).

### Deletion of *MoSEC4* leads to defects in vegetative growth and conidiation

To study the function of MoSec4, we generated targeted gene replacement mutants of the *MoSEC4* gene in an *M. oryzae* parental strain Guy11_Δ*MoKu80* (hereinafter referred to as “Ku80”), which is wild type Guy11 with a deletion of the *MoKU80* gene to enhance homologous recombination (Villalba et al., 2008). Transformants were produced in which the ORF of the *MoSEC4* gene was replaced with a hygromycin B phosphotransferase (HPH) gene (Figure S1A). Two *MoSEC4* deletion mutants, Δ*Mosec4-11* and Δ*Mosec4-46*, were characterized by reverse transcription polymerase chain reaction (RT-PCR) and Southern blot analysis (Figures S1B,C). To confirm phenotypes associated with mutation of the *MoSEC4* gene, the entire ORF with its native promoter was introduced into the Δ*Mosec4-11* mutant (hereafter known as Δ*Mosec4*), and a complementation transformant Δ*Mosec4*-*Com* was also confirmed by Southern analysis (Figures S1B,C).

**Figure 2:**
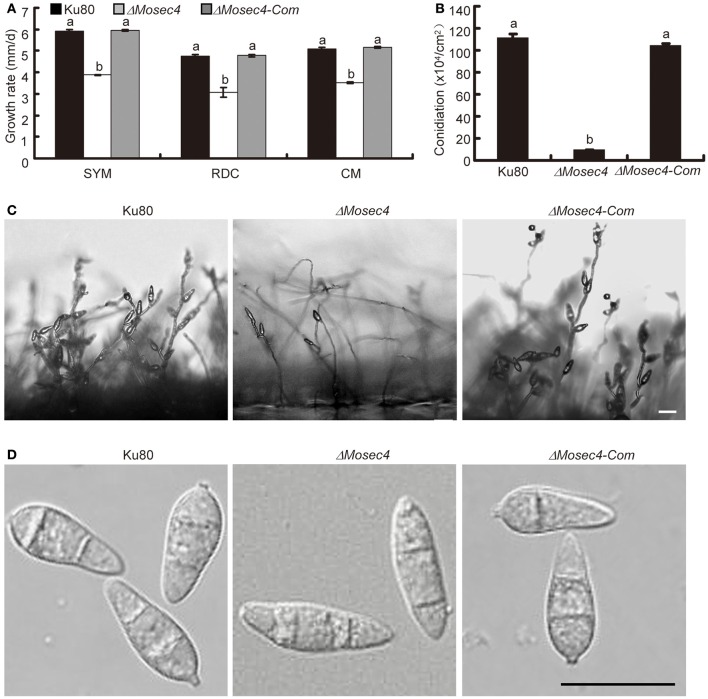
**The Δ*Mosec4* mutants showed defects in vegetative growth and conidial morphogenesis**. Results shown for Ku80, Δ*Mosec4* (Δ*Mosec4-11*), and Δ*Mosec4-Com* (the Δ*Mosec4-11* complemented strain) are from three independent experiments with standard deviations indicated. Similar hyphal growth and conidiation results were observed for independent mutant Δ*Mosec4-46*. **(A)** Diameter of hyphal growth at 10 days after incubation on starch yeast medium (SYM), rice bran medium (RDC), and complete medium (CM) agar plates at room temperature. See Figure S2 for more details. **(B)** Numbers of conidia harvested from a 9 cm rice bran plate after incubation for 7 days at room temperature. **(C)** Conidial development was observed microscopically 24 h after induction of conidiation. Bar = 20 μm. **(D)** Differential interference contrast (DIC) microscopy of conidial morphology. Bar = 20 μm. The letters indicate statistically significant differences (*p* < 0.01).

To explore the role of MoSec4 in vegetative growth, the parental strain (Ku80), Δ*Mosec4* mutants and complemented mutant (Δ*Mosec4-Com*) were grown on SYM, RDC, and CM media. The Δ*Mosec4* mutants exhibited a significant reduction of growth rate on all the media (Figure 2A and Figure S2). In addition, the Δ*Mosec4* mutants displayed more branching and were prone to form swollen hyphal tips (Figure S3). Additionally, the fluorescence of the polarized growth marker MoExo70-GFP fusion was not observed at mutant hyphal tips (Figure S4). This suggested that filamentous hyphal tips frequently undergo depolarization in the mutants.

We also evaluated the role of MoSec4 in conidiation of *M. oryzae*. The results showed that the Δ*Mosec4* mutants produced significantly fewer conidia compared with the parental (Ku80) and Δ*Mosec4-Com* strains (Figure 2B). Consistent with this result, we observed that the number of conidia per conidiophore was reduced in Δ*Mosec4* mutants (Figure 2C). This result indicated that deletion of *MoSEC4* caused a defect in sympodial conidiogenesis and subsequent reduction of conidium production. Furthermore, conidia from the Δ*Mosec4* mutants displayed abnormal morphology. Specifically, mutant conidia lost the pyriform-shape and showed a higher length-width ratio than those of the parental and complemented strains (Figure 2D and Table S1). Taken together, these results suggested that MoSec4 plays a role in conidiogenesis of *M. oryzae.*

### *ΔMosec4* mutants are more sensitive to various stressors

**Figure 3:**
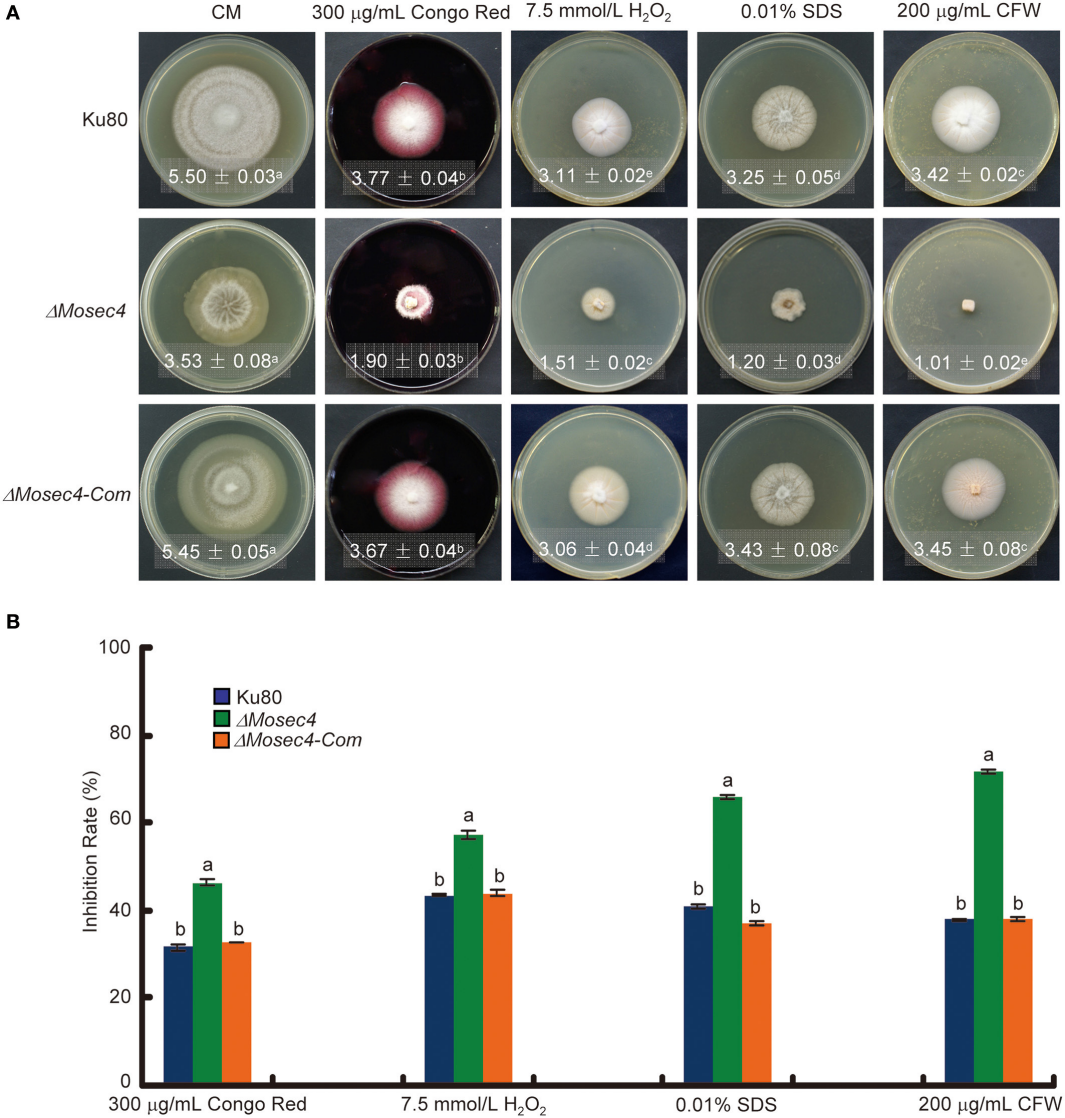
**TheΔ*Mosec4* mutants are more sensitive to cell wall-associated stresses. (A)** Strains were grown on CM medium without or with various stress inducers as indicated for 9 days at room temperature, then photographed. Colony diameters with standard deviations are shown in each panel. The letters indicate statistically significant differences (*p* < 0.01). **(B)** Colony diameters were subjected to statistical analysis. The growth inhibition rate is relative to the growth rate of each untreated control. Inhibition rate = (diameter of untreated strain-diameter of treated strain)/(diameter of untreated strain) × 100%. Three replications were performed with similar results. The letters indicate statistically significant differences (*p* < 0.01).

In *S. cerevisiae*, deletion of *Sec4p* leads to the abnormal deposition of chitin, which is an important component of fungal cell walls. To determine if MoSec4 is involved in response to stresses reported to be associated with cell wall integrity, we investigated the growth of Δ*Mosec4* mutants on media containing each of several stress-inducing chemicals, including Congo Red, H_2_O_2_, SDS, and CFW. Among them, Congo Red binds to glucan and acts as a cell wall perturbing agent (Wood and Fulcher, 1983; Song et al., 2010). SDS is a detergent used to test cell wall integrity, because a defect in cell wall integrity will increase the accessibility of SDS to damage the plasma membrane (Shimizu et al., 1994; Igual et al., 1996; Bickle et al., 1998). CFW binds to nascent chitin and glucan (with less affinity), perturbs the microfibril assembly and subsequently impairs the cell wall integrity (Elorza et al., 1983; Ram et al., 1994; Lussier et al., 1997). Additionally, H_2_O_2_ causes oxidative stress (Bai et al., 2003). Growth of Δ*Mosec4* mutants on media containing Congo Red, H_2_O_2_, SDS, and CFW was severely inhibited, especially on media with CFW (Figure 3). These results suggested that MoSec4 may be involved in response to different stresses, including stresses associated with integrity of hyphal cell walls.

### Deletion of *MoSEC4* attenuates pathogenicity on susceptible rice

**Figure 4:**
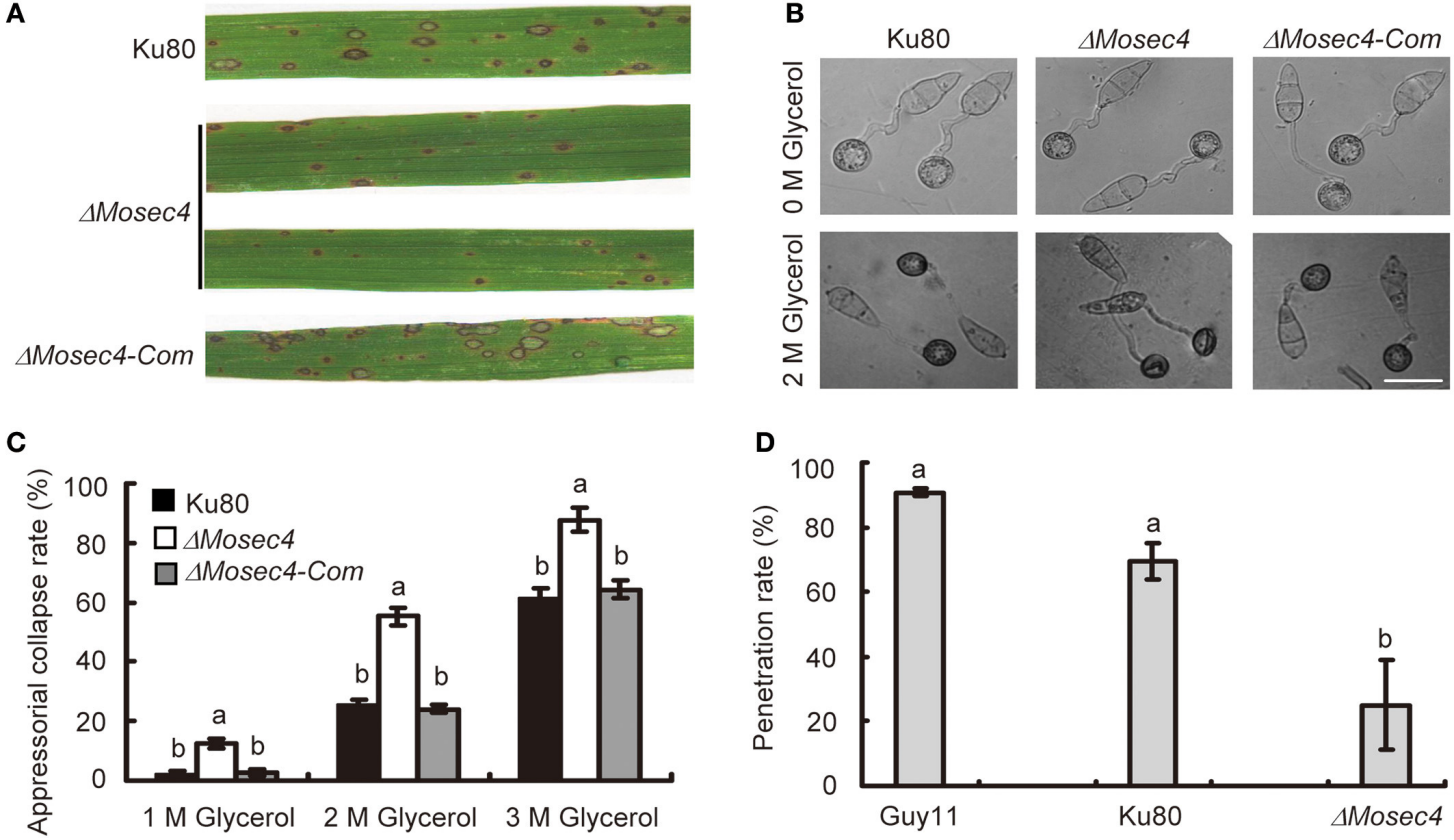
**Assessment of pathogenicity and appressorial turgor pressure of the Δ*Mosec4* mutants. (A)** Leaves of rice cultivar CO39 were inoculated with conidial suspensions of Ku80, Δ*Mosec4*, and Δ*Mosec4-Com*. Typical leaf symptoms were photographed at 7 days after inoculation. The parental and complemented strains produce predominantly Type 3 (up to 1.5 mm long) and Type 4 (up to 2 mm) susceptible lesions with green to tan centers capable of abundant sporulation (Figure S5A). In contrast the mutant produced predominantly Type 1 lesions (up to 0.5 mm long), which are enlarged, uniformly dark brown resistance spots that fail to sporulate. The mutant also produced fewer lesions than nonmutant strains (Figure S5B). **(B)** The percent of appressorial collapse after incubation in different concentrations of glycerol indicates their relative turgor pressures in a standard cytorrhysis assay. Appressorial collapse is illustrated here for 2 molar glycerol. Bar = 20 μm. **(C)** A cytorrhysis assay indicates the *MoSEC4* appressoria are defective in building turgor pressure required for host penetration. Appressoria at 24 h post germination were treated with different concentrations of glycerol (1M, 2M, 3M), and examined by microscopy 5 min later. For each glycerol concentration, at least 100 appressoria were observed and numbers of collapsed appressoria were counted. Results are from three independent experiments with standard deviations. The letters indicate statistically significant differences (*p* < 0.01). **(D)** Penetration rate on leaf sheath at 28 hpi. At least 100 appressoria were observed and numbers of infection hyphae were counted for each strain. The letters indicate statistically significant differences (*p* < 0.01).

To investigate whether MoSec4 was involved in pathogenicity, conidial suspensions of Ku80, Δ*Mosec4*, and Δ*Mosec4-Com* strains were inoculated respectively, onto 3-week-old rice seedlings of susceptible rice cultivar CO39. At 7 days post inoculation (dpi), the parental strain and complemented transformant caused Type 3 to Type 4 lesions with green/tan centers that are capable of abundant sporulation (Valent et al., 1991). In contrast, the Δ*Mosec4* mutants produced Type 1 hypersensitive flecks that fail to sporulate or Type 2 lesions with small green/tan centers and limited sporulation potential (Figure 4A and Figure S5A). Moreover, the number of lesions produced by the Δ*Mosec4* mutants was decreased (~50%) relative to the parental strain and complemented mutant (Figure S5B).

The attenuated pathogenicity of Δ*Mosec4* mutants could be caused by a defect in growth, infection-related development and/or the secretion of effectors. In order to understand what caused the defect, we first assessed the effects of *MoSEC4* deletion on germination of conidia as well as formation and maturation of appressoria. Deletion of *MoSEC4* did not affect the conidial germination, and it retarded, but did not block appressorial formation (Figure S6). Therefore, neither process appears responsible for the observed pathogenicity defect.

We next examined the turgor pressure in appressoria of parental strain Ku80 and Δ*Mosec4* mutants using a standard cytorrhysis assay, in which appressoria collapse when the external glycerol concentration exceeds the concentration of intracellular osmolytes, such as glycerol (de Jong et al., 1997). The results showed that, after treatment with 2 M glycerol for 5 min, only 25% of appressoria produced by the parental or complemented strains collapsed, whereas, under the same conditions, more than 60% of appressoria produced by the Δ*Mosec4* mutant collapsed (Figures 4B,C). Therefore, appressoria produced by the mutant had lower levels of turgor pressure, which could account for lower levels of host cuticle penetration and lower numbers of lesions in leaf infection assays.

**Figure 5:**
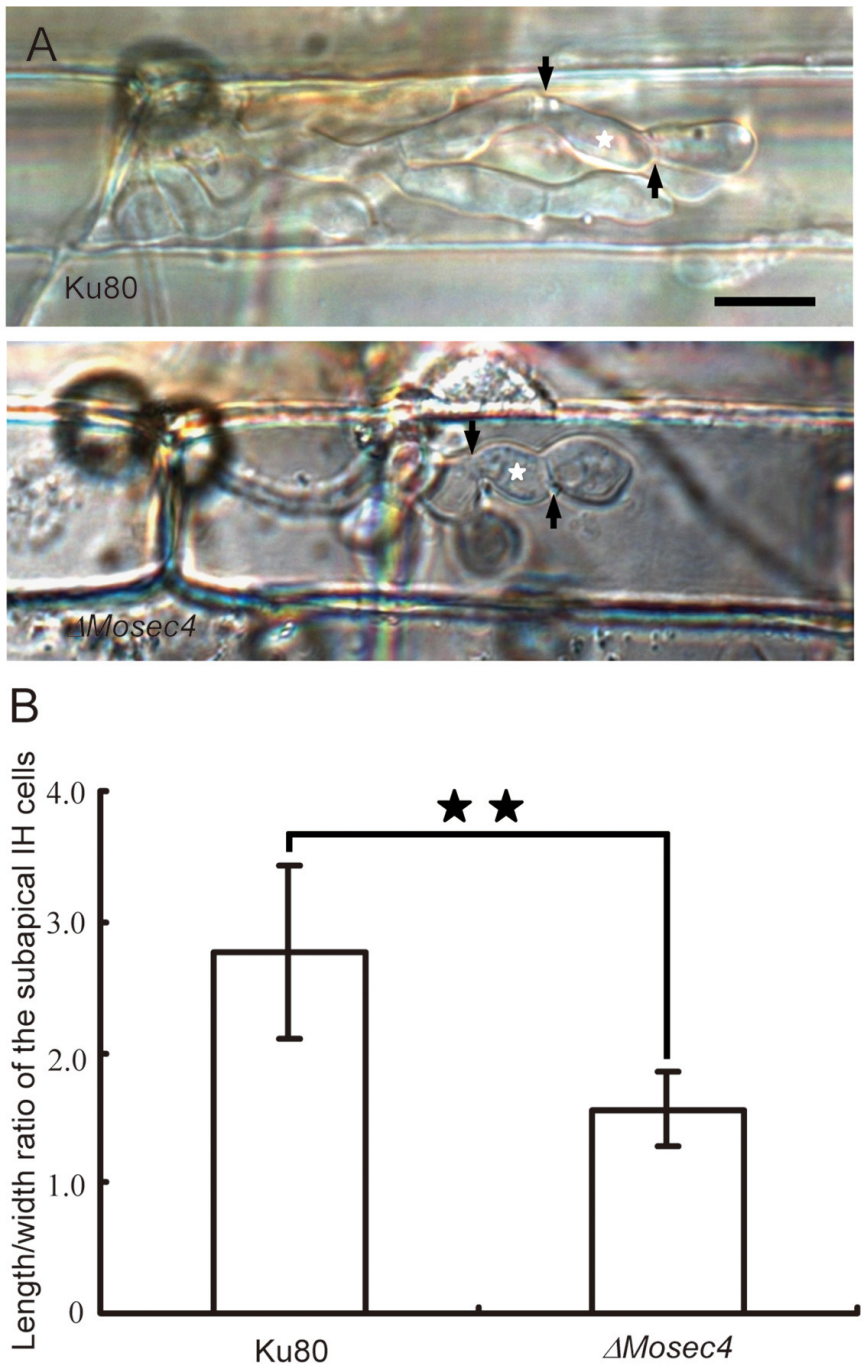
**Invasive hyphal cell shape is altered in the Δ*Mosec4* mutant. (A)** Typical morphology of IH in the Ku80 and Δ*Mosec4* mutant. The black arrows indicate septa. The white stars indicate the subapical bulbous hyphae subjected to the length to width ratio assay. Bar = 20 μm. **(B)** Statistical analysis of the length to width ratio of the subapical IH cells in Ku80 and the Δ*Mosec4* mutant. The stars indicate a significant difference between the Ku80 and Δ*Mosec4* mutant (*P* < 0.01).

We also performed a leaf sheath inoculation assay to document the appressorial penetration rates and biotrophic invasion of Guy11, Ku80, and Δ*Mosec4* mutants on susceptible rice YT16. Consistently, the penetration rate of the Δ*Mosec4* mutant at 28 hpi was ~25% compared with Guy11, which is significantly reduced compared to penetration rates of ~70% for Ku80 compared to Guy11 (Figure 4D). Penetration by the mutant strain was ~36% of the rate of penetration by the parental strain Ku80. Live-cell imaging was performed to observe the morphology of IH produced by the Δ*Mosec4* mutant inside rice cells. The results showed that the individual bulbous IH cells of the Δ*Mosec4* mutant were often shorter and swollen, which makes the mutant IH appear compressed (Figure 5A). For the Δ*Mosec4* mutant, 91% of the IH (110 of 121 randomly imaged IH) had shorter and swollen bulbous IH cells, compared to 2.7% of those for Ku80 (2 of 74 randomly imaged IH). A detailed analysis of the length to width ratio of the subapical bulbous IH cells (location indicated in Figure 5A) showed that the average length to width ratio of tested cells is 1.56 for Δ*Mosec4* mutant IH cells, which is significantly less than the ratio of 2.77 for Ku80 (Figure 5B). Thus, there is a morphological effect on mutant IH, which could potentially impact leaf colonization and lesion development.

### MoSec4 localized at the tip region in the growing hyphae

**Figure 6:**
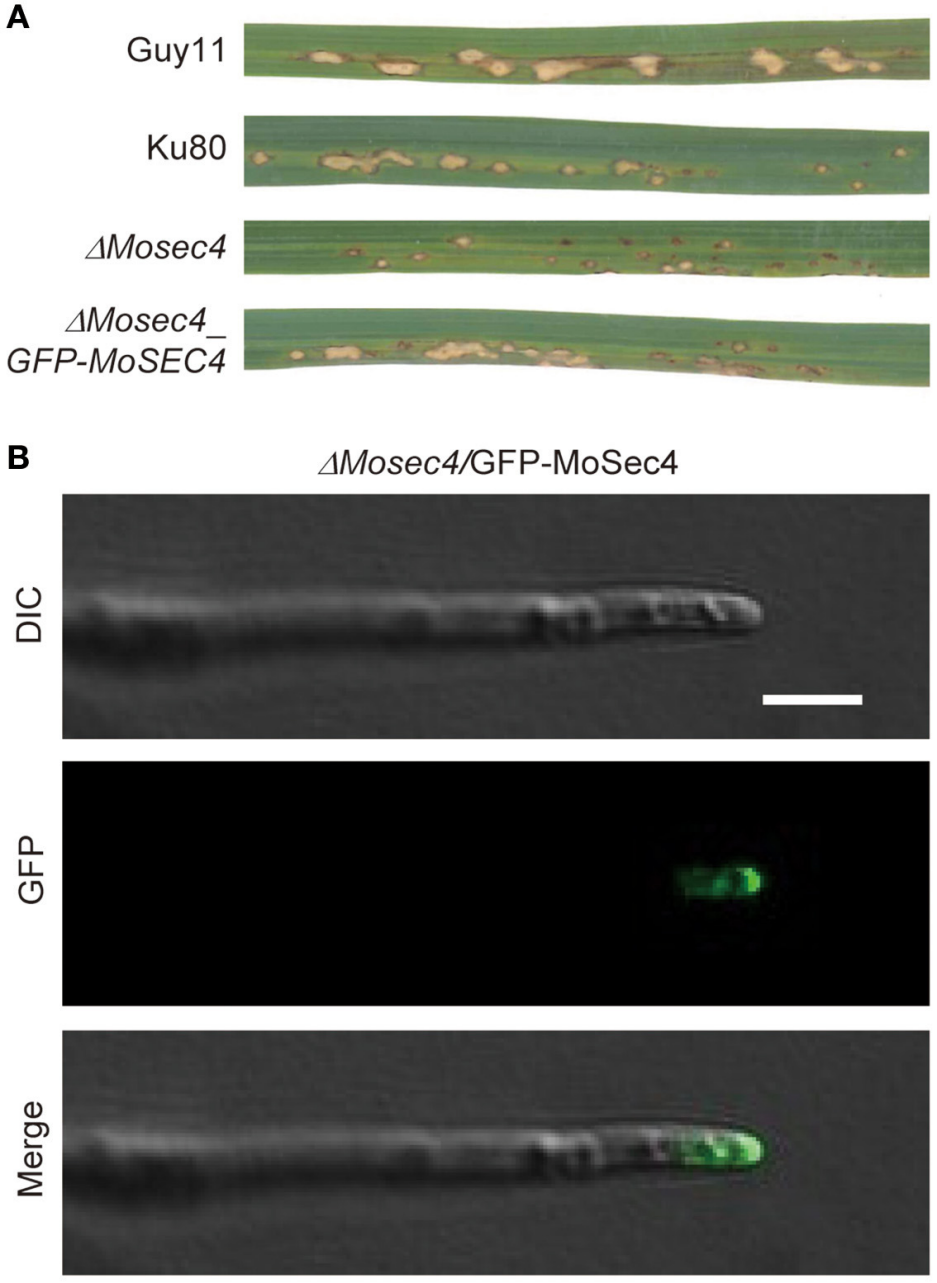
**The GFP-MoSec4 fusion protein mainly accumulated at the apical region of growing hyphal tips. (A)** Expression of the GFP-MoSec4 fusion under the control of the native promoter could rescue the attenuated pathogenicity of Δ*Mosec4* mutant. The GFP-MoSec4 complementation strain produced larger lesions characteristic of the Ku80 strain in rice cultivar YT16 at 7 dpi. **(B)** The GFP-MoSec4 fusion mainly localized at growing hyphal tips. Bar = 5 μm.

To investigate the localization of MoSec4 protein, the *MoSEC4* gene was expressed with its native promoter and an N-terminal GFP fusion (*MoSEC4p:GFP:MoSEC4*) and introduced into the Ku80 and Δ*Mosec4* strains (Figure S7A). The phenotype of Δ*Mosec4*_*GFP-MoSEC4* transformants was analyzed to assess whether the GFP-MoSec4 fusion protein was functional. Our microscopy results showed that the expression of GFP-MoSec4 fusion could restore wild type spore shape (Figure S7B and Table S1). The GFP-MoSec4 fusion protein also complemented the attenuated pathogenicity of Δ*Mosec4* mutant (Figure 6A). Genotyping confirmed the insertion of the *P*_*MoSEC*4_*:GFP:MoSEC4* fusion in all the transformants (Figure S7C). Taken together, these results suggested that the GFP-MoSec4 fusion protein is functional and again confirms that defects described in the mutant are caused by deletion of *MoSEC4* gene.

Live-cell imaging of growing vegetative hyphae was performed for localization of the GFP-MoSec4 fusion protein. The fluorescent MoSec4 protein mainly localized in crescents adjacent to the tips of growing hyphae (Figure 6B), which is consistent with a conserved function in secretion of hyphal cell wall components and apical growth.

### MoSec4 is required for the secretion of extracellular enzymes

**Figure 7:**
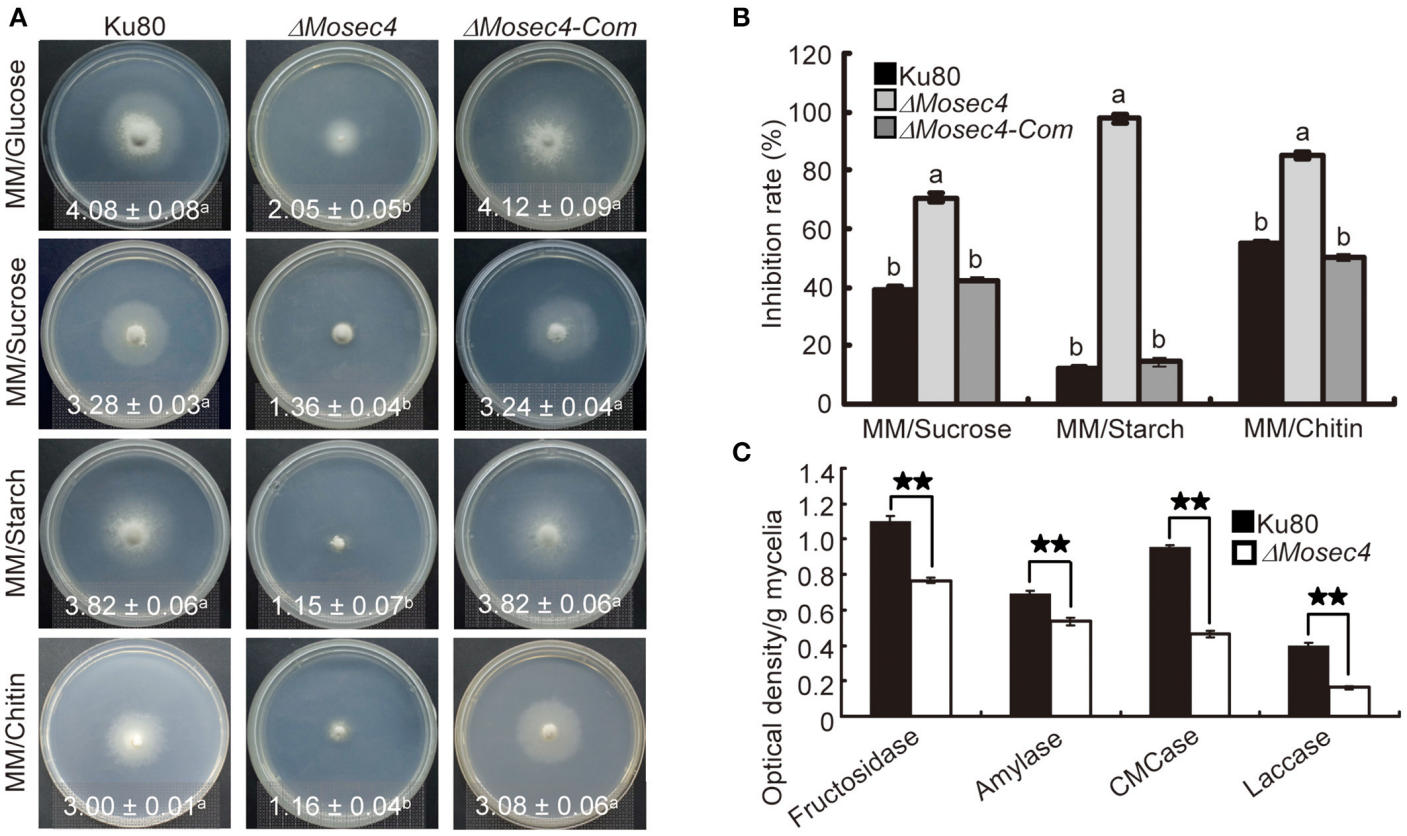
**The Δ*Mosec4* mutant appears defective in the secretion of extracellular enzymes. (A)** Vegetative growth on MM media supplemented with a 1% carbon source, including glucose, sucrose, starch, and chitin. Colony diameters at 10 days after inoculation are shown in each panel with standard deviations. The letters indicate statistically significant differences (*p* < 0.01). **(B)** Statistical analysis of the growth inhibition rate of mycelia grown on different MM media. Inhibition rate = (Diameter of growth on glucose-Diameter of growth on polysaccharide)/(Diameter of growth on glucose) × 100%. The letters indicate statistically significant differences (*p* < 0.01). **(C)** Enzymatic activities of CMCase, fructosidase, amylase, and laccase were detected using culture filtrates from the wild type and the Δ*Mosec4* mutant grown in CM liquid medium. The absorbance of fructosidase, amylase and CMCase was determined at A520 nm, and laccase at A420 nm. Enzyme concentrations = optical density per gram dry weight of mycelium. The stars indicate statistically significant differences (*p* < 0.01).

To determine whether MoSec4 is required for the secretion of extracellular enzymes, we cultured both the Ku80 and the Δ*Mosec4* mutant on MM media containing different disaccharides or polysaccharides as the sole carbon source to examine the secretion of relevant hydrolases. We found that the mycelial growth of Δ*Mosec4* mutants was dramatically inhibited (Figure 7A). When grown on the MM media containing sucrose, starch, or chitin, the mycelial growth rate of Δ*Mosec4* mutants was inhibited by 36, 48, and 46%, respectively, compared to growth on glucose (Figure 7B). These results suggested that *MoSEC4* deletion partially blocked the secretion of several extracellular hydrolases, invertase, amylolytic enzyme and chitinase (respectively involved in the enzymolysis of sucrose, starch, and chitin) and subsequently inhibited the utilization of these carbon sources. Furthermore, we directly determined the activity of carboxymethyl cellulase (CMCase), fructosidase and amylase in fungal culture filtrates, and the results showed that the activity of these extracellular enzymes was dramatically decreased for the Δ*Mosec4* mutant (Figure 7C). Taken together, our data suggest that MoSec4 plays an important role in the secretion of extracellular enzymes.

### Mutation in *MoSEC4* partially disrupted the localization of cytoplasmic effectors

**Figure 8:**
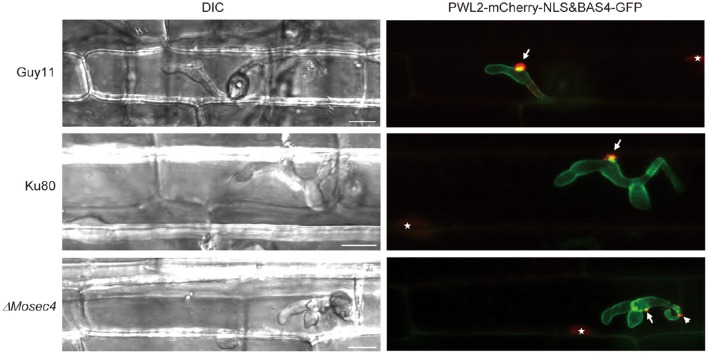
**Localization of cytoplasmic effector to BICs was partially disrupted in the Δ*Mosec4* mutant**. The apoplastic effector fusion BAS4-GFP and cytoplasmic effector fusion PWL2-mCherry-NLS, engineered with a C-terminal nuclear localization signal (NLS) to concentrate translocated effector protein in the rice nucleus, were introduced into GUY11, the Ku80 and the Δ*Mosec4* mutant. Transformants were inoculated into the leaf sheath of susceptible rice YT16. White arrows indicate BICs, white arrowhead indicates the BIC-like structure, and white stars indicate PWL2-mCherry-NLS fluorescence in rice nuclei. Bars = 20 μm.

To assess whether the MoSec4 protein is also involved in the secretion of effectors, we performed live cell imaging of biotrophic invasion by mutant strains expressing cytoplasmic effector fusion protein PWL2-mCherry-NLS (with an added nuclear localization signal “NLS” to facilitate detection of translocated effector protein in rice cells), and apoplastic effector fusion BAS4-GFP (Khang et al., 2010). As expected, in the Guy11 and Ku80 strains, the BAS4-GFP fusion protein outlines the IH and occupies an inner layer of the BIC, and the PWL2-mCherry-NLS protein mainly concentrated in BICs and was translocated into rice nuclei. In contrast, the localization of PWL2-mCherry-NLS for the Δ*Mosec4* mutant was partially disrupted, while localization of the BAS4-GFP fusion appeared normal (Figure 8). Localization patterns of PWL2-mCherry-NLS in Δ*Mosec4* mutant could be further divided into three groups as shown in Figure S8. Among all the observations, 34.1% of the randomly imaged infection sites (30 in 88) were normal (type I), 39.8% (35 in 88) of them formed additional small fluorescent punctae outside of BICs (type II), and 26.1% (23 in 88) of them formed two or more BIC-like structures (type III). In contrast, only 15.6% (5 in 32) of the randomly imaged infection sites in Ku80 showed the type II pattern, and none showed type III. Most of the Ku80 sites appeared normal. Therefore, proper localization of cytoplasmic effectors in BICs depends in part on MoSec4.

## Discussion

In this study, all identified MoSec4 mutant phenotypes as well as GFP-MoSec4 localization as a fluorescent crescent adjacent to growing hyphal tips were consistent with a role in extracellular protein secretion, as shown for Sec4/Rab8 orthologs in diverse eukaryotic organisms. The Δ*Mosec4* mutants are altered in vegetative hyphal morphology and growth, conidial morphology and production, secretion of extracellular enzymes, and sensitivity to stress treatments. During plant invasion, the mutants build-up less appressorial turgor pressure required for host cuticle penetration, and they show a defect in penetration. Biotrophic invasive hyphal morphology and cytoplasmic effector secretion patterns are also altered. Abnormal hyphal morphology indicates a defect in targeting of secretion of cell wall components to required growth points, consistent with a regulatory role for MoSec4.

The Δ*Mosec4* mutants showed reduced pathogenicity toward rice, producing fewer lesions as well as smaller lesions with greatly reduced potential for sporulation. The pathogenicity defect doesn't appear to be due to the misshapen mutant conidia, which germinate normally. Melanized appressoria are formed by the mutant, although slightly more slowly than by the parental strain. However, compared to parental strain Ku80, ~36% of mutant appressoria penetrated the leaf sheath cuticle and developed IH in first invaded cells. Our finding that mutant appressoria build up less turgor pressure than appressoria from nonmutant strains could at least partially account for the penetration defect. Mutant IH that do form inside first-invaded rice cells are often misshapen. Specifically, these IH appear more compact because mutant bulbous IH cells are shortened (sometimes almost round) compared to individual bulbous IH cells of the parental strain. Finally, focused accumulation of cytoplasmic effectors into BICs often appears abnormal. These multiple factors, including both morphological defects and impaired secretion patterns, could together account for the pathogenicity defect in the Δ*Mosec4* mutants.

Although extracellular protein secretion pathways are best understood for the budding yeast *S. cerevisiae*, increasing research is being conducted on filamentous fungi, including *Neurospora cras*sa, *Aspergillus niger*, and *M. oryzae* (Punt et al., 2001; Jones and Sudbery, 2010; Riquelme et al., 2014; Guo et al., 2015; Gupta et al., 2015). For filamentous fungi, key secretion components are conserved, but they may play different roles to accommodate different lifestyles. Unlike Sec4p in budding yeast, as well as Sec4 in *Candida albicans* and Rab8 in mammalian cells (Salminen and Novick, 1987; Goud et al., 1988; Mao et al., 1999; Hattula et al., 2006), Sec4/Rab8 orthologs in filamentous fungi are not essential for survival, suggesting that there are partially redundant pathways in these fungi. To support rapid polarized hyphal tip growth by filamentous fungi, vesicles that deliver components for building new hyphal cell walls concentrate in the Spitzenkörper (SPK), a vesicle supply center where secretory vesicles accumulate before they are directed for secretion to the growing hyphal tip (Riquelme, 2013; Riquelme et al., 2014; Riquelme and Sánchez-Léon, 2014). In growing hyphae, the exocyst complex components, as well as the Sec4/Rab8 orthologs, localized to the space between the SPK and the hyphal tip (Jones and Sudbery, 2010; Pantazopoulou et al., 2014; Riquelme et al., 2014; Guo et al., 2015; Zheng et al., 2015). In *M. oryzae* vegetative hyphae, the SPK and the entire 8-component exocyst are conserved and localize to growing tips as expected (Giraldo et al., 2013; Gupta et al., 2015). We show that GFP-MoSec4 protein also localized as a crescent-shaped structure at vegetative hyphal tips, and that the typical Exo70-GFP localization pattern in vegetative hyphae is not observed in the Δ*Mosec4* mutants. This is consistent with MoSec4 playing a role in maintaining apical tip extension associated with hyphal growth in *M. oryzae*.

We set out to determine if MoSec4p plays a regulatory role in the nonconventional, exocyst-mediated secretion of cytoplasmic effectors into BICs. This was predicted because in *S. cerevisiae*, Sec4p plays a key role in assembly and recruiting of the exocyst complex for its role in tethering secretory vesicles to the target membrane. Then SNARE proteins mediate vesicle fusion to the PM and release of vesicle contents to the extracellular space. The involvement of the exocyst in efficient secretion of cytoplasmic effectors was documented because fluorescent effector fusion proteins were partially retained inside BIC-associated cells in Δ*Mosec5* and Δ*Moexo70* mutants (Giraldo et al., 2013). However, we did not observe cytoplasmic effectors retained inside BIC-associated cells of the Δ*Mosec4* mutant, as was observed for the exocyst component mutants. Indeed, cytoplasmic effector mislocalization patterns after secretion by the Δ*Mosec4* mutant were more reminiscent of the “double BIC” phenotype associated with the Δ*Mosso1* mutant (Giraldo et al., 2013). Therefore, MoSec4 may not be a major regulator involved in exocyst-mediated secretion of cytoplasmic effectors. However, it might play some role in proper BIC development. Notably, the Sec4 ortholog in *C. orbiculare* has been reported to be involved in the secretion of cytoplasmic effectors (Irieda et al., 2014), although there is currently no evidence for a nonconventional effector secretion pathway in this system.

We identified both similarities and differences between MoSec4 mutant phenotypes and those in different filamentous fungi. Vegetative hyphae of the *M. oryzae* Δ*Mosec4* mutant have more branches and swollen vegetative hyphal tips, similar to mutants with defects in *Bcsas1* of *B. cinerea* (Zhang et al., 2014) and *srgA* of *A. niger* (Punt et al., 2001). However, the Δ*Mosec4* mutant was more sensitive to hydrogen peroxide while the *BcSas1* deletion mutant was less sensitive to hydrogen peroxide (Zhang et al., 2014). Additionally, mutation of Sec4/Rab8 orthologs can result in a conditional restriction of protein secretion in filamentous fungi. For instance, the SrgA protein in *A. niger* was required for protein secretion on glucose but not on maltodextrin as carbon source (Punt et al., 2001). The Δ*Mosec4* phenotypes from this study are partial phenotypes. For example, the mutant appressorial penetration rate on susceptible rice plants varied from 12 to 39.6% of the penetration rate of Guy11. One possible explanation is that one or more bypass pathways could partially complement the secretion defect under certain growth conditions. Taken together, the Sec4/Rab8 orthologs may play different roles in development and stress responses in different organisms and under different conditions.

Sec4 orthologs in *M. oryzae* and other fungal pathogens are required not only for vegetative growth, but also for pathogenicity (Siriputthaiwan et al., 2005; Powers-Fletcher et al., 2013; Zhang et al., 2014; Zheng et al., 2015). It remains possible that the pathogenicity defect is in part due to general stress from a defect in protein trafficking impacting general fitness of the fungus. Indeed, we found that the mutant grows less well on glucose than the parental strain, which is consistent with a general fitness defect. However, the increased branching and hyphal tip depolarization phenotypes associated with mutant hyphae could also account for slower growth on glucose. Reduced growth of the Δ*Mosec4* mutant on media with sucrose, starch and chitin as sole carbon source suggests impaired secretion of enzymes needed for utilization of these nutrient sources. Direct enzyme assays showed that Δ*Mosec4* vegetative hyphae secrete less cellulase, fructosidase, amylase, and laccase into culture medium than the parental strain. Laccase is an important virulence effector that could protect pathogens from the toxic phytoalexins and tannins (Mayer and Staples, 2002). Cumulative effects of impaired secretion at all infection stages would be expected to have a negative effect on pathogenicity.

Previously, insight into the function of Sec4 orthologs has been obtained for filamentous fungi, including plant pathogens, growing *in vitro*. For *M. oryzae*, live-cell imaging of the fungus invading rice cells has been yielding new insights on biotrophic invasion strategies including effector secretion and translocation. Mutation of MoSec4 has an interesting effect on IH morphology inside rice cells, potentially leading to new insight on how these special cells grow. Understanding the detailed molecular and cellular mechanisms of biotrophic invasion by the rice blast fungus is critical for achieving sustainable control of this globally devastating disease.

## Author contributions

JZ, ZW, and BV conceived and designed the experiments. HZ, SC, XC, SL, XD, CY, and MG performed the experiments. HZ, SM, XC and BV wrote the manuscript. JZ, ZW, BV and EO revised and approved the manuscript.

### Conflict of interest statement

The authors declare that the research was conducted in the absence of any commercial or financial relationships that could be construed as a potential conflict of interest.

## Supplementary material

Table S1 **The width to length ratio of the Δ*Mosec4* mutant spores**.

Table S2 **Plasmids used in this study**.

Table S3 **Primers used in this study**.

Figure S1 **Generation of Δ*Mosec4* mutants. (A)** Restriction map of the *MoSEC4* genomic region and deletion construct pG06135. Arrows indicate orientations of *MoSEC4* and the hygromycin phosphotransferase (*HPH*) genes. The *MoSEC4* deletion construct pG06135 contained the homologous sequences flanking the *HPH* gene to replace the *MoSEC4* ORF. **(B)** Total RNA samples (approximately 1 μg per reaction) isolated from mycelia of wild-type strain Ku80, and deletion mutants Δ*Mosec4-11* and Δ*Mosec4-46* were subjected to RT-PCR using *MoSEC4* gene-specific primers (Table S3). The RT-PCR product is a 377 bp fragment in Ku80 as predicted, but it is missing in the deletion mutants. **(C)** Total genomic DNA samples (5 μg per lane) isolated from Ku80, *MoSEC4* deletion mutants and complementation transformant Δ*Mosec4-Com* (derived from Δ*Mosec4-11*) were digested with *EcoR*I and subjected to Southern blot analysis using the *MoSEC4* ORF-specific primers (4F and 4R) to generate the probe (Table S3). A 5.0 kb target band was present only in Ku80 and Δ*Mosec4-Com* as predicted.

Figure S2 **The Δ*Mosec4* mutants displayed reduced vegetative growth**. Strains were grown on starch yeast (SYM), rice bran (RDC) and complete (CM) media for 10 days at room temperature, then photographed. The diameter of colony growth and standard deviation are shown in each panel. The letters indicate statistically significant differences (*p* < 0.01). Similar results were obtained in three independent biological repetitions.

Figure S3 **Hyphae ofΔ*Mosec4* mutants showed enhanced branching and swollen hyphal tips during growth on agar slides**. Hyphae of Ku80 **(A)** and the Δ*Mosec4* mutant **(B,C)** were imaged after germination and growth on water agar slides overnight. Typical nonbranched hyphae in **(A)** contrast with branched mutant hyphae in **(B)**. The insets from **(C)** are enlarged in **(D,E)** to show swollen mutant hyphal tips. Bars = 20 μm.

Figure S4 **MoExo70-GFP localization appears disrupted in the Δ*Mosec4* mutant**. The Exo70:GFP fusion protein was expressed in Ku80 (**left**) and the Δ*Mosec4* mutant (**right**). Spores of the transformants were germinated on agar slides and growing hyphae were observed using the Zeiss LSM780 confocal microscope system. Bar = 5 μm.

Figure S5 **The Δ*Mosec4* mutant produces leaf lesions that are smaller in size and fewer in number than nonmutant strains**. Leaves of rice cultivar CO39 were spray inoculated with conidial suspensions (1 × 10^5^ conidia/ml) of Guy11, Ku80, the Δ*Mosec4* mutant, and its complemented strain. **(A)** Quantification of different lesion types was performed as described in Figure 4A. **(B)** The number of lesions on 5 cm-long leaf pieces were counted after 7 days, focusing on the youngest leaf tissue at the time of inoculation. For each strain, at least 20 leaves were used for counting. The results are from three independent experiments with standard deviations. Note that similar numbers of lesions were produced by Guy11 and Ku80, although lesion expansion was reduced in Ku80 (Type 4, up to 2 mm) relative to Guy11 (Type 5, up to 3 mm). See also Figure 6A. The letters indicate statistically significant differences (*p* < 0.01).

Figure S6 **Conidial germination and appressorial formation by the *MoSEC4* deletion mutant**. Conidial suspensions of Ku80 and Δ*Mosec4* mutant were applied on the hydrophobic side of Gelbond film as described in Section Materials and Methods, then examined with DIC microscopy. At least 100 conidia were counted. Results are from three independent experiments with standard deviations. The letters indicate statistically significant differences (*p* < 0.01).

Figure S7 **The GFP-MoSec4 fusion protein was functional and could complement the spore shape defect of the Δ*Mosec4* mutant. (A)** Graphic presentation of GFP-MoSec4 fusion construct. **(B)** The expression of GFP-MoSec4 fusion under control of the native promoter could rescue the spore shape defect in the Δ*Mosec4* mutant. Bar = 20 μm. **(C)** A PCR-based genotyping of Guy11 (1), Ku80 (2), Δ*Mosec4* (3), Ku80_*GFP-MoSEC4* (4), and Δ*Mosec4_GFP-MoSEC4* transformants (5–9).

Figure S8 **Different *in planta* localization patterns for cytoplasmic effector fusion PWL2-mCherry (mCH) -NLS and apoplastic BAS4-GFP fusions in Δ*Mosec4* mutant**. The localization of PWL2-mCherry-NLS fusion could be classified into three types. Type I appeared normal. Type II exhibited additional small fluorescent punctae, in addition to BIC localization. Type III appeared to form two or more BIC-like structures. The arrows indicated the BIC and BIC-like structure. The arrow heads indicated the small punctae. Bar = 10 μm.
