# Supplementary material for: The Small GTPase MoSec4 Is Involved in Vegetative Development and Pathogenicity by Regulating the Extracellular Protein Secretion in Magnaporthe oryzae
Source: Front Plant Sci. 2016 Sep 27;7:1458. doi: 10.3389/fpls.2016.01458 (PMC5037964; doi:10.3389/fpls.2016.01458)
Supplement: Table S1 — The width to length ratio of the ΔMosec4 mutant spores. [file Table1.PDF]

Table S1 The width to length ratio of the *ΔMosec4* mutant spores.

Strain Name	Length/Width
Ku80	2.26 ± 0.15[b]
ΔMosec4	2.90 ± 0.37[a]
ΔMosec4_GFP-MoSEC4	2.30 ± 0.31[b]
