# Supplementary material for: The Small GTPase MoSec4 Is Involved in Vegetative Development and Pathogenicity by Regulating the Extracellular Protein Secretion in Magnaporthe oryzae
Source: Front Plant Sci. 2016 Sep 27;7:1458. doi: 10.3389/fpls.2016.01458 (PMC5037964; doi:10.3389/fpls.2016.01458)
Supplement: Table S2 — Plasmids used in this study. [file Table2.PDF]

Table S2 Plasmids used in this study.

| Clone | Description |
|---|---|
| pBV1160 | For expression of PWL2-mCherry-NLS and BAS4-GFP fusion, the *PWL2-mCherry-NLS & BAS4-GFP* fusion was amplified from pBV591 with PBV591SacIF and PBV591SalIR, and inserted into the *Sac*I-*Sal*I sites of pBV403. Kanamycin and Geneticin (G418) resistance. |
| pBV1171 | For expression of MoSec4-GFP fusion, the *MoSEC4* promoter, *GFP* cds and *MoSEC4* cds were amplified and digested with *Spe*I-*Xba*I, *Xba*I-*BamH*I, *Bam*HI-*Sbf*I respectively, and inserted into the *Spe*I-*Pst*I sites of pBV579. The $P_{MoSEC4}$*::GFP;MoSEC4* fusion was cut from the intermediate vector with *Mfe*I and *Sal*I, and inserted into the *Eco*RI-*Sal*I sites of pBV403. Kanamycin and Geneticin (G418) resistance. |
| pG06135KO | For *MoSEC4* knock out, The downstream and upstream fragment of *MoSEC4* were amplified from the Guy11 genomic DNA using primers *MoSEC4*-2F & *MoSEC4*-2R, and *MoSEC4*-3F & *MoSEC4*-3R. The purified products were digested with *Hin*d III and *Eco*R I, *Bam*H I and *Spe* I respectively, and inserted into the *Hin*d III- *Eco*R I, *Bam*H I-*Spe* I sites of pCX62. Ampicillin and Hygromycin resistance. |
| pG06135COM | For *ΔMosec4* complementation, a 2.2-kb genomic DNA fragment containing the native promoter, entire ORF and 3'-UTR of *MoSEC4* was amplified using primers *MoSEC4*-6F and *MoSEC4*-6R and inserted into plasmid pKNTG. Ampicillin and Geneticin (G418) resistance. |
| pBV947 | For expression of Exo70:GFP, 2.97 kb of Exo70 gene, including 1 kb promoter and coding sequence, cloned in EcoRI-BamHI sites of pBV403. Kanamycin and G418 resistance (Giraldo et al., 2013). |
