# Supplementary material for: The Small GTPase MoSec4 Is Involved in Vegetative Development and Pathogenicity by Regulating the Extracellular Protein Secretion in Magnaporthe oryzae
Source: Front Plant Sci. 2016 Sep 27;7:1458. doi: 10.3389/fpls.2016.01458 (PMC5037964; doi:10.3389/fpls.2016.01458)
Supplement: Table S3 — Primers used in this study. [file Table3.PDF]

Table S3 Primers used in this study.

**Primer**	**Sequence (5’ -3’)**	**Enzyme cutting Site**
MoSEC4-2F	CCCAAGCTTGCGTAGGTGGCAGACAGA	*Hind*III
MoSEC4-2R	CCGGAATTCCGCGGAGGATGATTATGG	*Eco*RI
MoSEC4-3F	GCGGGATCCGCTGCTAGAGGAAGATGG	*Bam*HI
MoSEC4-3R	GGACTAGTGATTAGAAGGACGGACGA	*Spe*I
MoActin-F	GCTGTCCTCGTCGATCTCGA	
MoActin-R	CAGAGCAGGTCAGGTAACGA	
MoSEC4-4F	TCTTTCACGCCGTCATTC	
MoSEC4-4R	TTGATGTTGCCCTTAGCC	
MoSEC4-5F	CACGGTATGCTTTGTCAGTC	
MoSEC4-5R	GACAGACGTCGCGGTGAGTT	
MoSEC4-6F	CCCAAGCTTCGAGTTCACCCGAATGTA	*Hind*III
MoSEC4-6R	CCGGAATTCGACCGAATATCTCAGAGGAT	*Eco*RI
*Sec4pSpeI*F	CAACTAGTCAATTGCTGGAAGATCTTGAGGACGTGGTG	*Spe*I
*Sec4pXbaI*R	ATTCTAGACATGGTGCTGCGTTTGGAGCAGC	*Xba*I
*GFPXbaI*F	TGTCTAGAATGGTGAGCAAGGGCGAGGAG	*Xba*I
*GFPBamHI*R	ATGGATCCCTTGTACAGCTCGTCCATGCCGAG	*BamH*I
*Sec4BamHI*F	AGGGATCCATGGCCAACAGGAATTACGATG	*BamH*I
*Sec4SbfI*R	AACCTGCAGGCTAGCAGCACTTTCCGCCCATGC	*Sbf*I
*pBV591SacI*F	CCGAGCTCTAACAATTTCACACAGGAAACAGC	*Sal*I
*pBV591SalI*R	TCGTCGACCAGGGTTTTCCCAGTCACGACGT	*Sal*I
MoSEC4ptF	TTATTGGCGCTGCCGTTTCGA	
MoSEC4tR	TGATGGTGCGGAAGCGTTCCT	
