# Supplementary figures and images for: The Small GTPase MoSec4 Is Involved in Vegetative Development and Pathogenicity by Regulating the Extracellular Protein Secretion in Magnaporthe oryzae
Source: Front Plant Sci. 2016 Sep 27;7:1458. doi: 10.3389/fpls.2016.01458 (PMC5037964; doi:10.3389/fpls.2016.01458)

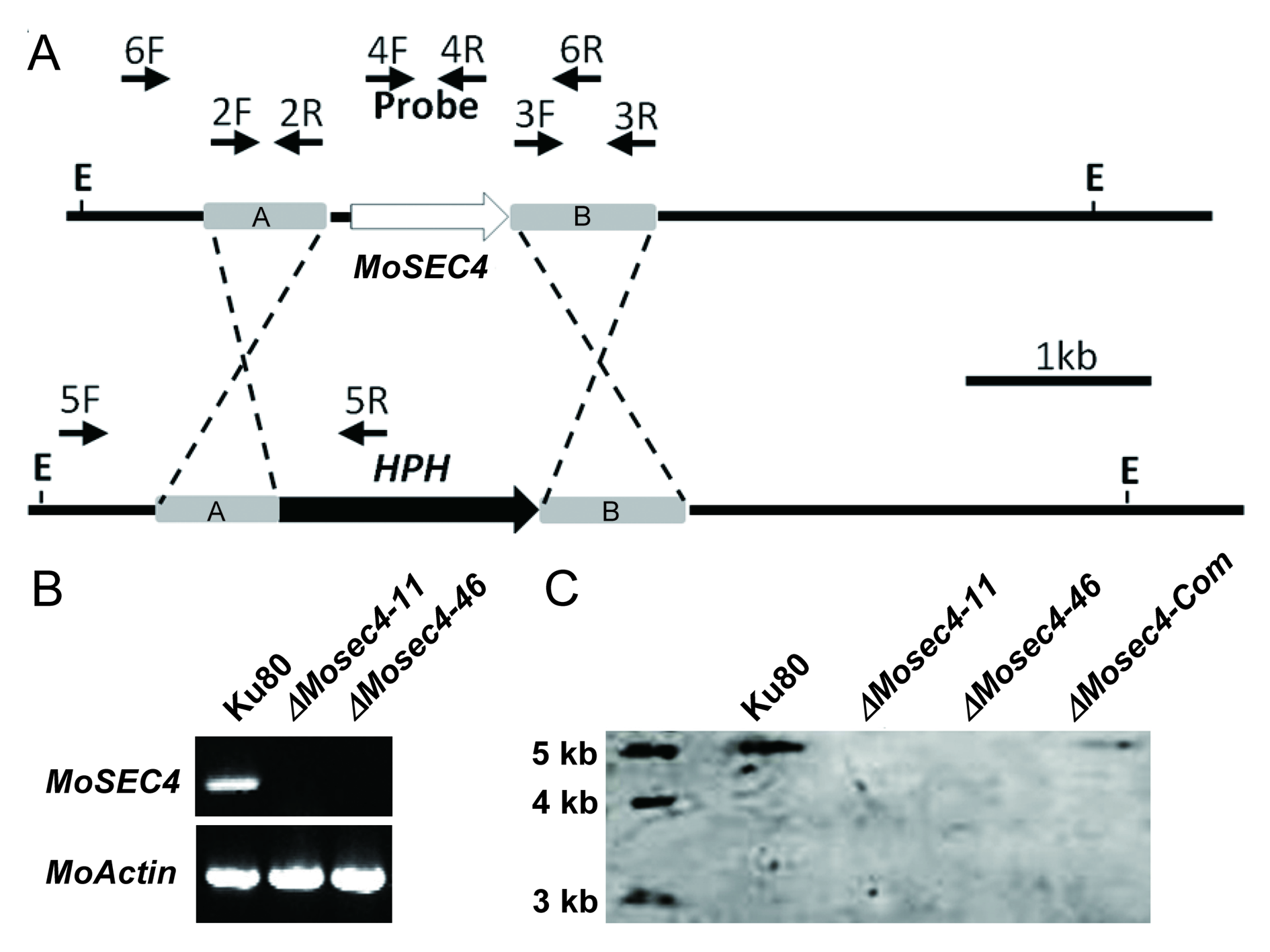

Supplement: Figure S1 — Generation of ΔMosec4 mutants. (A) Restriction map of the MoSEC4 genomic region and deletion construct pG06135. Arrows indicate orientations of MoSEC4 and the hygromycin phosphotransferase (HPH) genes. The MoSEC4 deletion construct pG06135 contained the homologous sequences flanking the HPH gene to replace the MoSEC4 ORF. (B) Total RNA samples (approximately 1 μg per reaction) isolated from mycelia of wild-type strain Ku80, and deletion mutants ΔMosec4-11 and ΔMosec4-46 were subjected to RT-PCR using MoSEC4 gene-specific primers (Table S3). The RT-PCR product is a 377 bp fragment in Ku80 as predicted, but it is missing in the deletion mutants. (C) Total genomic DNA samples (5 μg per lane) isolated from Ku80, MoSEC4 deletion mutants and complementation transformant ΔMosec4-Com (derived from ΔMosec4-11) were digested with EcoRI and subjected to Southern blot analysis using the MoSEC4 ORF-specific primers (4F and 4R) to generate the probe (Table S3). A 5.0 kb target band was present only in Ku80 and ΔMosec4-Com as predicted. [file Image1.TIF]

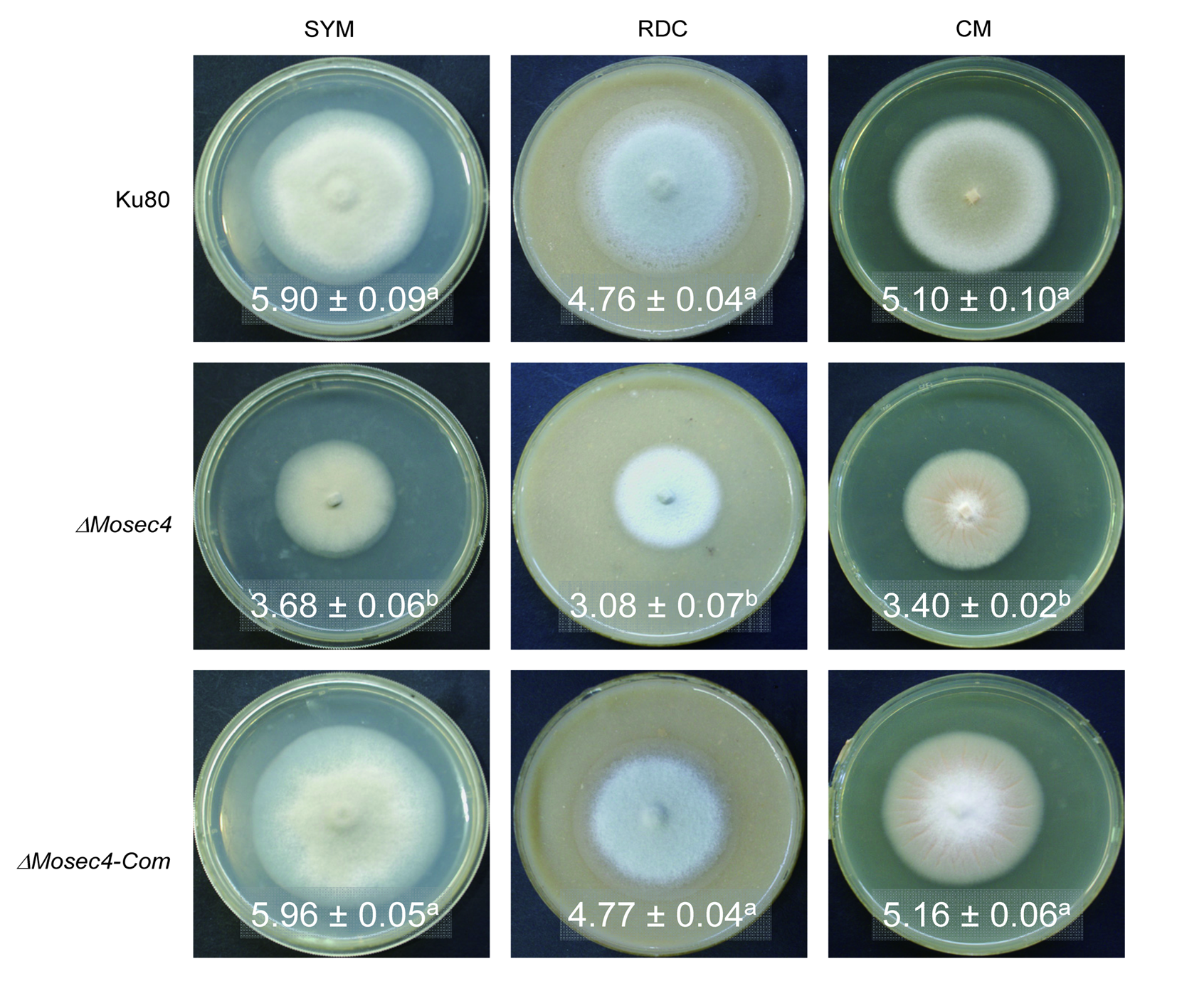

Supplement: Figure S2 — The ΔMosec4 mutants displayed reduced vegetative growth. Strains were grown on starch yeast (SYM), rice bran (RDC) and complete (CM) media for 10 days at room temperature, then photographed. The diameter of colony growth and standard deviation are shown in each panel. The letters indicate statistically significant differences (p < 0.01). Similar results were obtained in three independent biological repetitions. [file Image2.TIF]

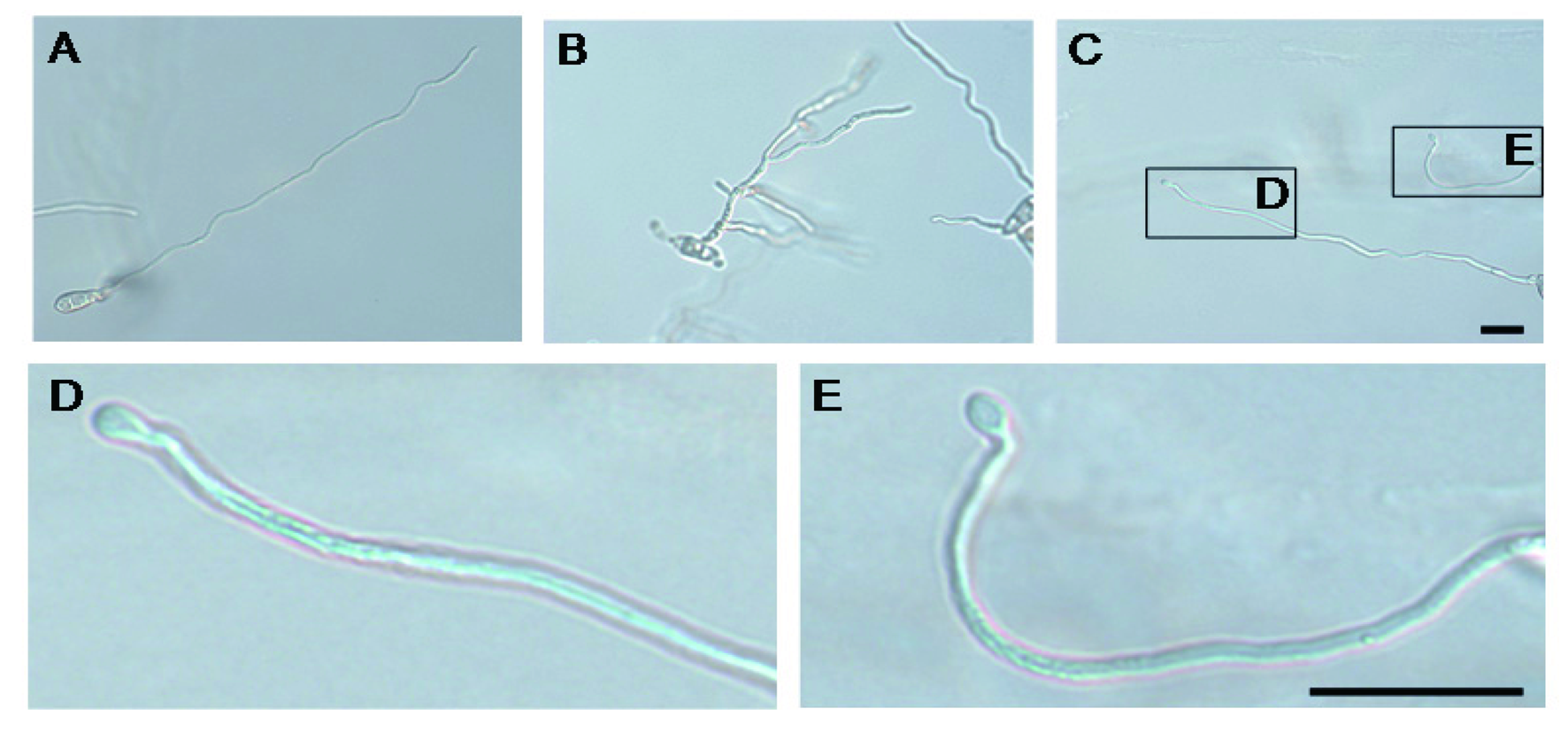

Supplement: Figure S3 — Hyphae ofΔMosec4 mutants showed enhanced branching and swollen hyphal tips during growth on agar slides. Hyphae of Ku80 (A) and the ΔMosec4 mutant (B,C) were imaged after germination and growth on water agar slides overnight. Typical nonbranched hyphae in (A) contrast with branched mutant hyphae in (B). The insets from (C) are enlarged in (D,E) to show swollen mutant hyphal tips. Bars = 20 μm. [file Image3.TIF]

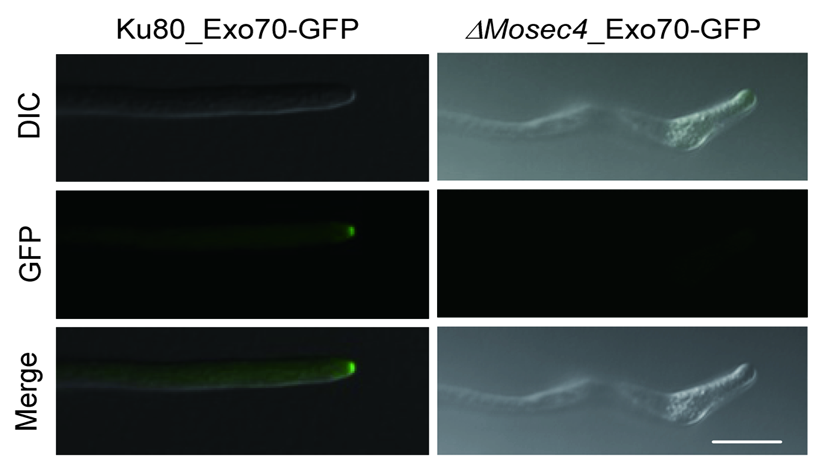

Supplement: Figure S4 — MoExo70-GFP localization appears disrupted in the ΔMosec4 mutant. The Exo70:GFP fusion protein was expressed in Ku80 (left) and the ΔMosec4 mutant (right). Spores of the transformants were germinated on agar slides and growing hyphae were observed using the Zeiss LSM780 confocal microscope system. Bar = 5 μm. [file Image4.TIF]

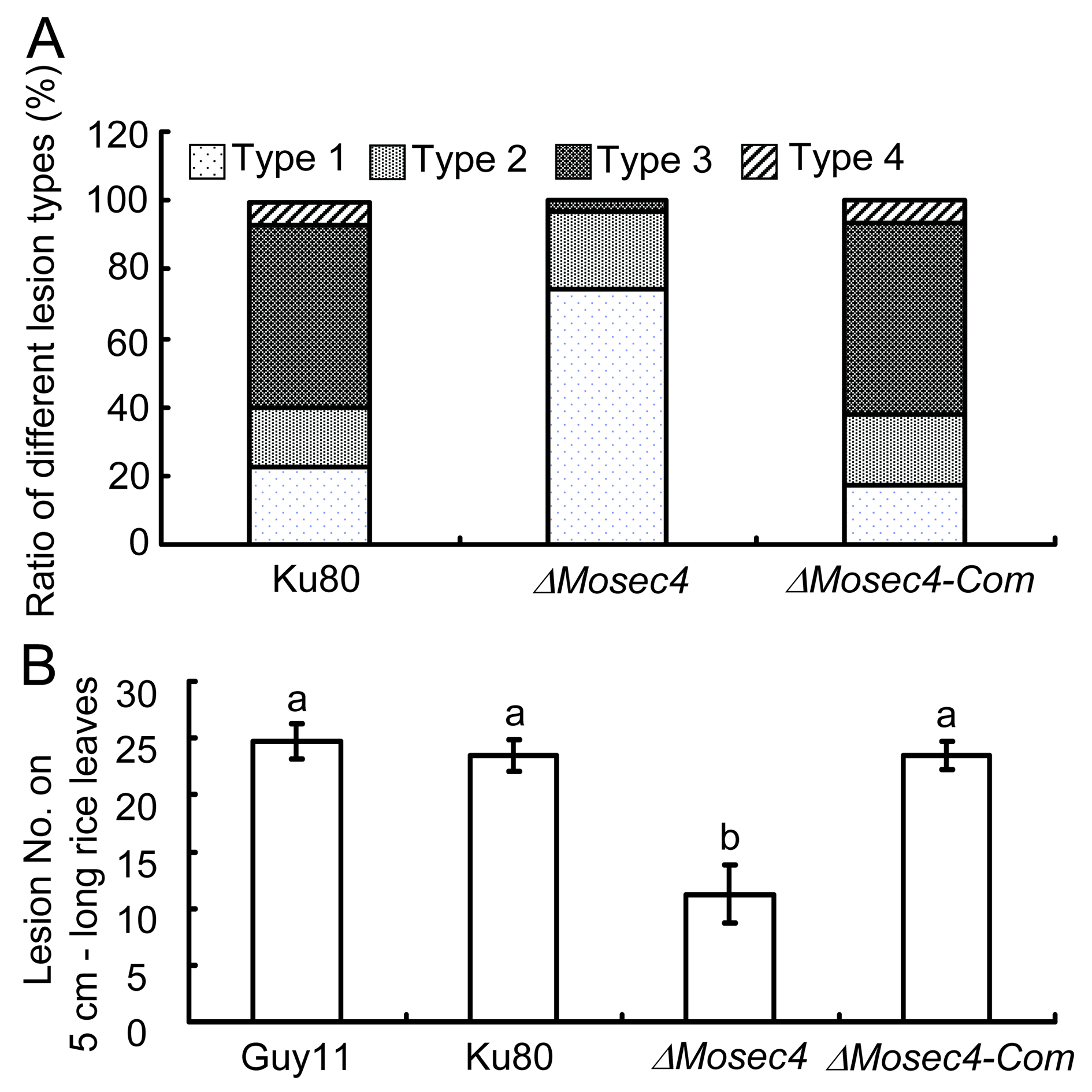

Supplement: Figure S5 — The ΔMosec4 mutant produces leaf lesions that are smaller in size and fewer in number than nonmutant strains. Leaves of rice cultivar CO39 were spray inoculated with conidial suspensions (1 × 10^5^ conidia/ml) of Guy11, Ku80, the ΔMosec4 mutant, and its complemented strain. (A) Quantification of different lesion types was performed as described in Figure 4A. (B) The number of lesions on 5 cm-long leaf pieces were counted after 7 days, focusing on the youngest leaf tissue at the time of inoculation. For each strain, at least 20 leaves were used for counting. The results are from three independent experiments with standard deviations. Note that similar numbers of lesions were produced by Guy11 and Ku80, although lesion expansion was reduced in Ku80 (Type 4, up to 2 mm) relative to Guy11 (Type 5, up to 3 mm). See also Figure 6A. The letters indicate statistically significant differences (p < 0.01). [file Image5.TIF]

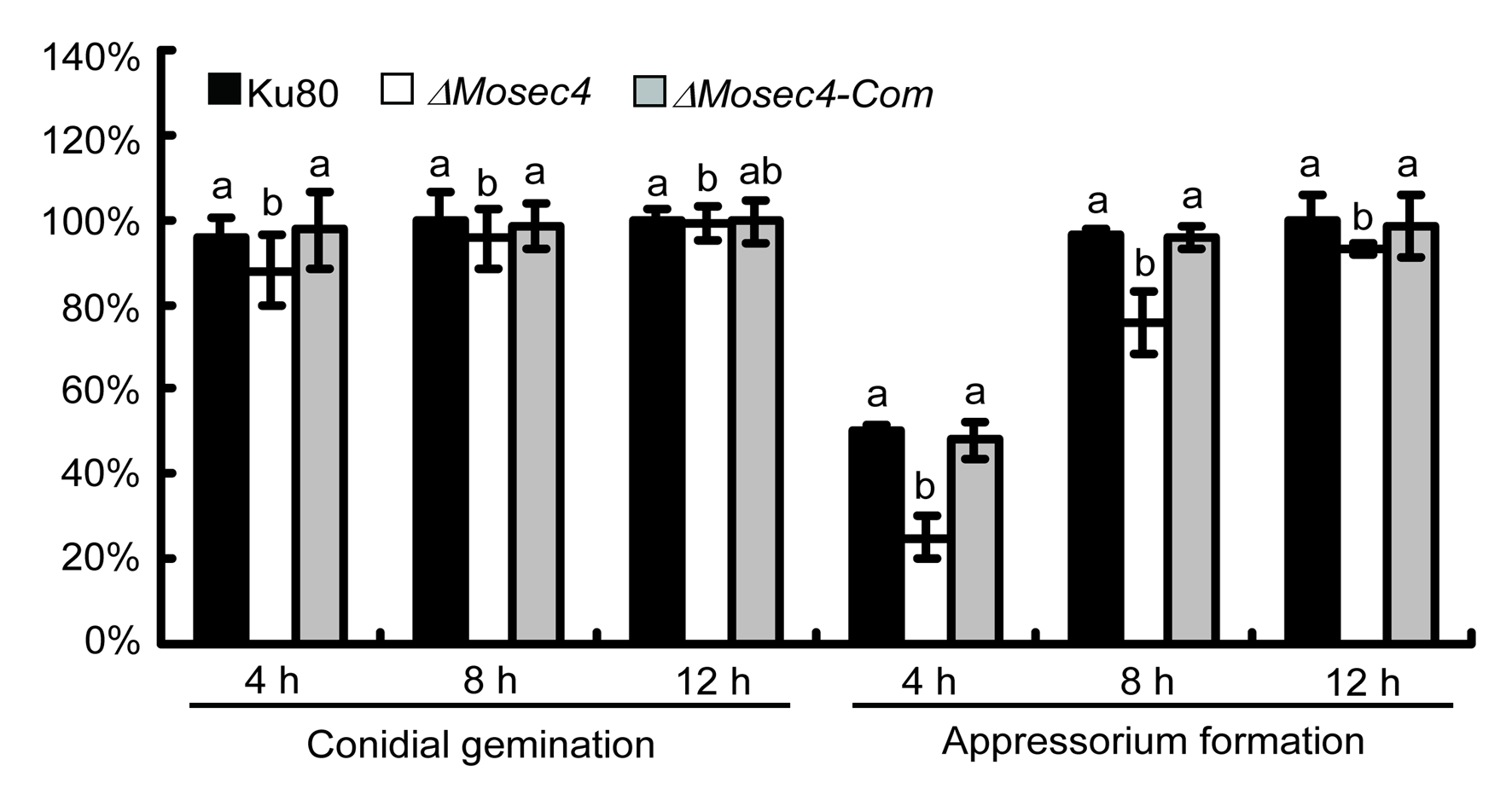

Supplement: Figure S6 — Conidial germination and appressorial formation by the MoSEC4 deletion mutant. Conidial suspensions of Ku80 and ΔMosec4 mutant were applied on the hydrophobic side of Gelbond film as described in Section Materials and Methods, then examined with DIC microscopy. At least 100 conidia were counted. Results are from three independent experiments with standard deviations. The letters indicate statistically significant differences (p < 0.01). [file Image6.TIF]

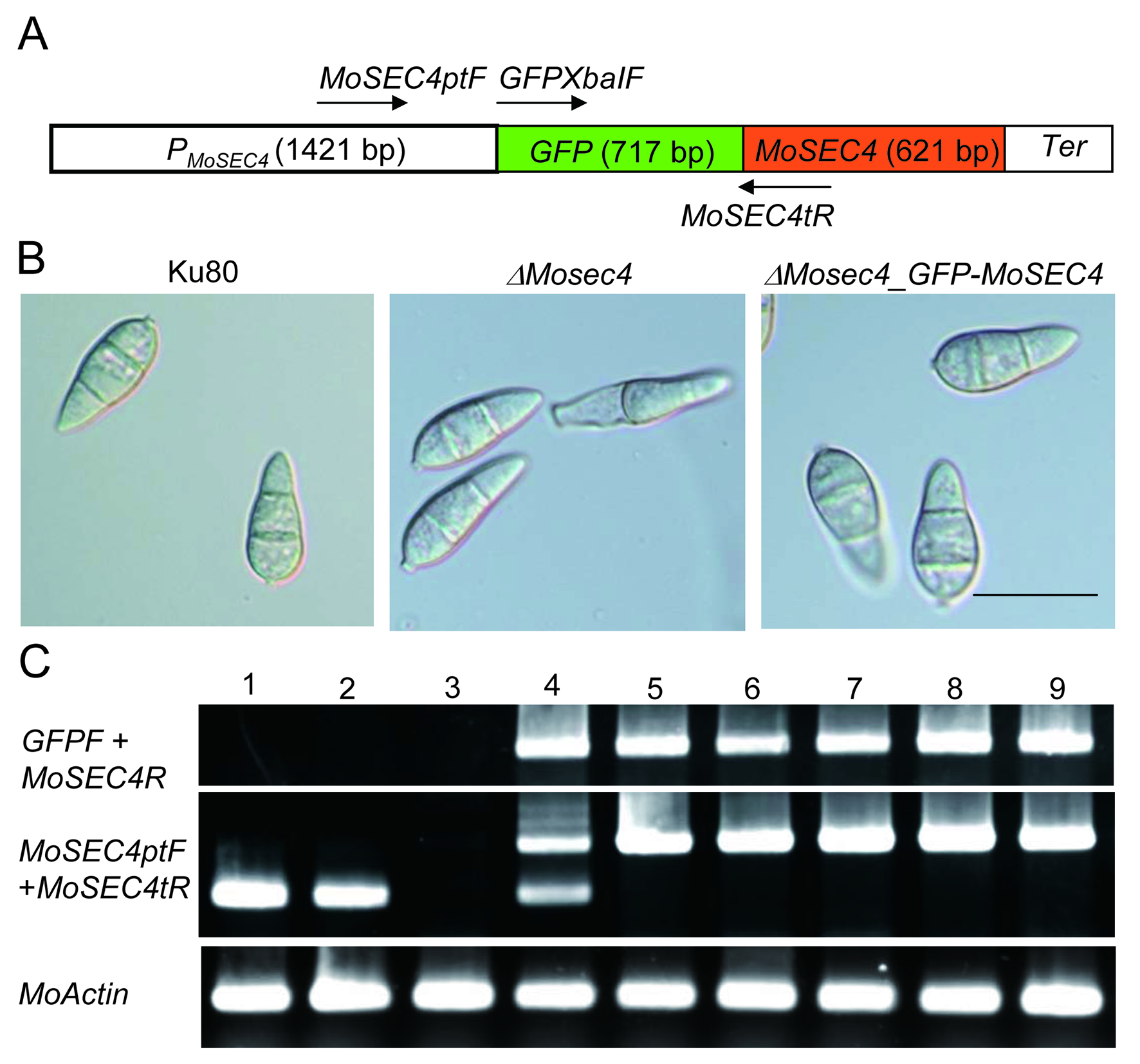

Supplement: Figure S7 — The GFP-MoSec4 fusion protein was functional and could complement the spore shape defect of the ΔMosec4 mutant. (A) Graphic presentation of GFP-MoSec4 fusion construct. (B) The expression of GFP-MoSec4 fusion under control of the native promoter could rescue the spore shape defect in the ΔMosec4 mutant. Bar = 20 μm. (C) A PCR-based genotyping of Guy11 (1), Ku80 (2), ΔMosec4 (3), Ku80_GFP-MoSEC4 (4), and ΔMosec4_GFP-MoSEC4 transformants (5–9). [file Image7.TIF]

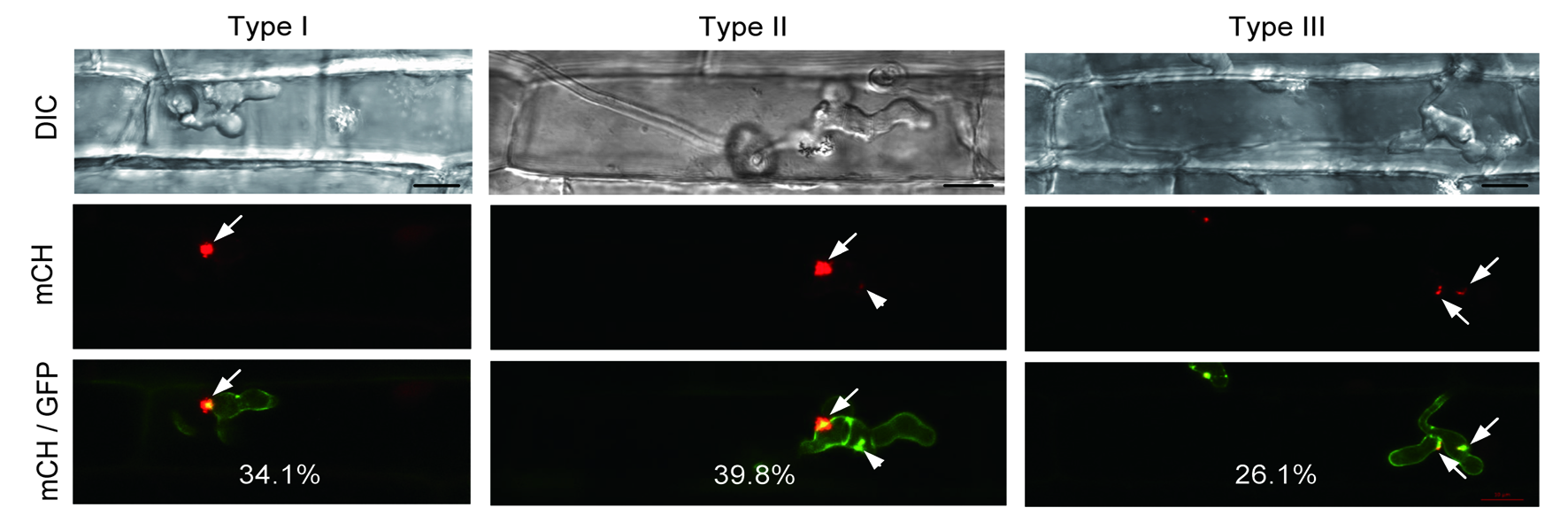

Supplement: Figure S8 — Different in planta localization patterns for cytoplasmic effector fusion PWL2-mCherry (mCH) -NLS and apoplastic BAS4-GFP fusions in ΔMosec4 mutant. The localization of PWL2-mCherry-NLS fusion could be classified into three types. Type I appeared normal. Type II exhibited additional small fluorescent punctae, in addition to BIC localization. Type III appeared to form two or more BIC-like structures. The arrows indicated the BIC and BIC-like structure. The arrow heads indicated the small punctae. Bar = 10 μm. [file Image8.TIF]
